# Evolution and expression analysis of the caffeoyl-CoA 3-*O*-methyltransferase (CCoAOMT) gene family in jute (*Corchorus* L*.*)

**DOI:** 10.1186/s12864-023-09281-w

**Published:** 2023-04-17

**Authors:** Mohamed Ali Kahie, Yongjun Wang, Pingping Fang, Jianmin Qi, Rongjie Lei, Jiantang Xu, Lihui Lin, Liwu Zhang, Jisen Zhang, Aifen Tao

**Affiliations:** 1 Key Laboratory of Ministry of Education for Genetics, Breeding and Multiple Utilization of Crops, Fujian Key Laboratory of Crop Breeding for Design, Fujian Agriculture and Forestry University, Fuzhou, 350002 China; 2 Center of Genomics & Biotechnology, Haixia Institute of Science & Technology, Fujian Agriculture and Forestry University, Fuzhou, 350002 China; 3 City University of Mogadishu, Mogadishu, 23111 Somalia; 4 State Key Lab for Conservation and Utilization of Subtropical AgroBiological Resources and Guangxi Key Lab for Sugarcane Biology, Guangxi University, Nanning, China

**Keywords:** Jute, *CCoAOMT*, Lignin, Evolution, Expression

## Abstract

**Background:**

Jute is considered one of the most important crops for fiber production and multipurpose usages. Caffeoyl-CoA 3-*O*-methyltransferase (CCoAOMT) is a crucial enzyme involved in lignin biosynthesis in plants. The potential functions of *CCoAOMT* in lignin biosynthesis of jute have been reported in several studies. However, little is known about the evolution of the *CCoAOMT* gene family, and either their expression level at different developing stages in different jute cultivars, as well as under abiotic stresses including salt and drought stress.

**Results:**

In the present study, 66 *CCoAOMT* genes from 12 species including 12 and eight *CCoAOMTs* in *Corchorus olitorius* and *C. capsularis* were identified. Phylogenetic analysis revealed that *CCoAOMTs* could be divided into six groups, and gene expansion was observed in *C. olitorius*. Furthermore, gene expression analysis of developing jute fibers was conducted at different developmental stages (15, 30, 45, 60, and 90 days after sowing [DAS]) in six varieties (Jute-179 [J179], Lubinyuanguo [LB], and Qiongyueqing [QY] for *C. capsularis*; Funong No.5 [F5], Kuanyechangguo [KY], and Cvlv [CL] for *C. olitorius*). The results showed that *CCoAOMT1* and *CCoAOMT2* were the dominant genes in the *CCoAOMT* family. Of these two dominant *CCoAOMTs*, *CCoAOMT2* showed a constitutive expression level during the entire growth stages, while *CCoAOMT1* exhibited differential expression patterns. These two genes showed higher expression levels in *C. olitorius* than in *C. capsularis.* The correlation between lignin content and *CCoAOMT* gene expression levels indicated that this gene family influences the lignin content of jute. Using real-time quantitative reverse transcription PCR (qRT-PCR), a substantial up-regulation of *CCoAOMTs* was detected in stem tissues of jute 24 h after drought treatment, with an up to 17-fold increase in expression compared to that of untreated plants.

**Conclusions:**

This study provides a basis for comprehensive genomic studies of the entire *CCoAOMT* gene family in *C. capsularis* and *C. olitorius.* Comparative genomics analysis among the *CCoAOMT* gene families of 12 species revealed the close evolutionary relationship among *Corchorus*, *Theobroma cacao* and *Gossypium raimondii.* This study also shows that *CCoAOMTs* are not only involved in lignin biosynthesis, but also are associated with the abiotic stress response in jute, and suggests the potential use of these lignin-related genes to genetically improve the fiber quality of jute.

**Supplementary Information:**

The online version contains supplementary material available at 10.1186/s12864-023-09281-w.

## Background

Jute, one of the most important sources of natural fibers, provides approximately 80% of the world’s bast fiber output. In recent years, jute is considered as an important cash crop with multipurpose usages like paper and textile industries, herbal medicine, leafy vegetable, and renewable biofuel source, etc. [1, 2]. Although there are more than 100 *Corchorus* species in the Malvaceae family, only *C*. *capsularis* and *C*. *olitorius* are commercially cultivated [3].

Lignin is a heterogeneous polymer without a defined primary structure and has monolignol monomers as precursors [4]. Recent studies have indicated that lignin plays a crucial role in determining stem strength, cell wall structural integrity, pathogen resistance, and water transfer efficiency [5–7]. Lignin provides mechanical support for the growth of vertical land plants. Nonetheless, high lignin content has a negative effect on jute fiber separation as well as fiber fineness [2, 8]. In fact, the lignin content of jute fibers is comparatively high (~ 15%), which distinguishes them with other non-wood bast fibers such as ramie, hemp and flax fibers (< 5%). Islam et al. reported that *C*. *olitorius* fibers contain more lignin and less cellulose than those of *C*. *capsularis* [3]. Therefore, it is essential to identify the regulatory factors involved in plant lignin biosynthesis to improve our ability to grow plants with low lignin levels. Lignification in plants is influenced by a number of plant-related factors. Compared to flax, jute exhibits duplications in the lignin biosynthetic genes cinnamoyl-CoA reductase (CCR), 4-coumarate: CoA ligase (4CL), caffeic acid O-methyltransferase (COMT), and Caffeoyl-CoA 3-*O*-methyltransferase (CCoAOMT). Many studies have reported that transcriptionally co-expressed genes are functionally associated and might interact with each other at the molecular and physiological levels. Monolignols are the precursors of lignin, which are synthesized as a branch of the phenylpropanoid pathway in the cytoplasm near the ER membrane and then transported to the cell wall to get polymerized into lignin [2, 9]. There are more than 10 gene families associated with monolignol biosynthesis, among which, *CCoAOMT* is one of the most important genes for monolignol biosynthesis [4, 10]. Chakraborty et al. discovered a total of 43 isoforms of all ten genes involved in monolignol biosynthesis of jute, including five isoforms for *CCoAOMT* [2]. The enzyme* CCoAOMT* is a S-adenosyl-L-methionine methyltransferase (SAM) central to the lignin biosynthetic pathway [5]. This protein is involved in the biosynthesis of lignin in plants as it catalyzes the methylation of caffeoyl CoA, an important metabolite for the biogenesis of guaiacyl lignin [11, 12]. The role of *CCoAOMT* in lignification was also supported by the fact that overexpression of jute *CCoAOMT* in *A. thaliana* resulted in the effect of increasing lignin content [13], and inhibition of *CCoAOMT* activity in transgenic tobacco significantly reduced lignin content in that species [14, 15]. Therefore, it is crucial to determine the expression of *CCoAOMT* genes in plants. Chakraborty et al. found *CcCCoAOMT2* was down-regulated in mutant bast tissues at an early growth stage of jute, while it was over-expressed at later growth stage [2]. Liu et al. investigated changes in the expression of *CCoAOMT* in loquat fruits stored at low temperatures, and found that the expression of this gene in green loquat fruits was lower than that in mature fruits [16]. Low temperature storage stimulated its transcription at the beginning of the storage process; however, its expression decreased throughout the rest of the storage period [16]. Zhang et al. reported that *CCoAOMT* is expressed in leaf tissues, roots, stem bark, and stems of jute, with an obviously greater transcription in the stem [13]. *CCoAOMT* expression was found to be particularly associated to all lignifying tissues, including phloem fibers, xylem fibers, and tracheary elements (TEs) in the stems of forsythia [17], tobacco [18], alfalfa [19–21], tomato [22], and soybean [23]. The existence of an intimate connection between lignification and levels of *CCoAOMT* transcripts has been reported in dicot crops [17], supporting the hypothesis that *CCoAOMT*-mediated methylation is commonly implied in the biosynthesis of lignin during normal growth and development of dicot plants.

Water deficiency occurs in plants whenever water provision is inadequate for development, transpiration, and photosynthesis [24], and ultimately leads to yield reduction [25]. Alvarez et al. reported that polyethylene glycol (PEG) treatment inhibited lignin biosynthesis in maize [25]. In the current context of global climatic change, water deficit stress is a major problem limiting production and productivity of jute. It causes 20% ~ 30% loss of fiber yield and also deteriorates the quality [26]. Meanwhile, salt stress negatively affects jute growth and physiological parameters, which subsequently reduces yield and quality [27]. Liu et al. studied the levels of *CCoAOMT* transcripts under abiotic stress in switchgrass and observed substantial up-regulation of this gene in stem tissues, as its expression increased by 33-fold in comparison to that of untreated plants [28]. The study of *CCoAOMT* expression under abiotic stress in switchgrass and other plants showed that this gene can be greatly impacted by drought stress [29]. However, the effects of NaCl and PEG stresses on the expression of jute *CCoAOMT* genes have not been thoroughly examined.

Phylogenetic analysis of sorghum demonstrated that one sorghum CCoAOMT-like enzyme is closely related to ancestral cyanobacterial CCoAOMT-like proteins. The remaining CCoAOMT-like enzymes, including those highly expressed in the leaves and stem, are closely related to the *CCoAOMT* gene family. Genes from these two groups have dissimilar or conserved gene structures [30]. Conversely, the identification and evolutionary analysis of *CCoAOMT* genes in jute have not yet been reported, and their transcriptional patterns were seldom studied. In order to determine the evolution of *CCoAOMT* gene family in jute, and to investigate the expression pattern of *CCoAOMT* genes in different situations of jute, we analyzed the evolution and expression patterns of members of the *CCoAOMT* gene family in the stem of distinct varieties of jute at different developmental stages, and revealed the levels of transcription of these genes under abiotic stress.

## Results

### Identification and cloning of *CCoAOMT* genes in jute

**Fig. 1:**
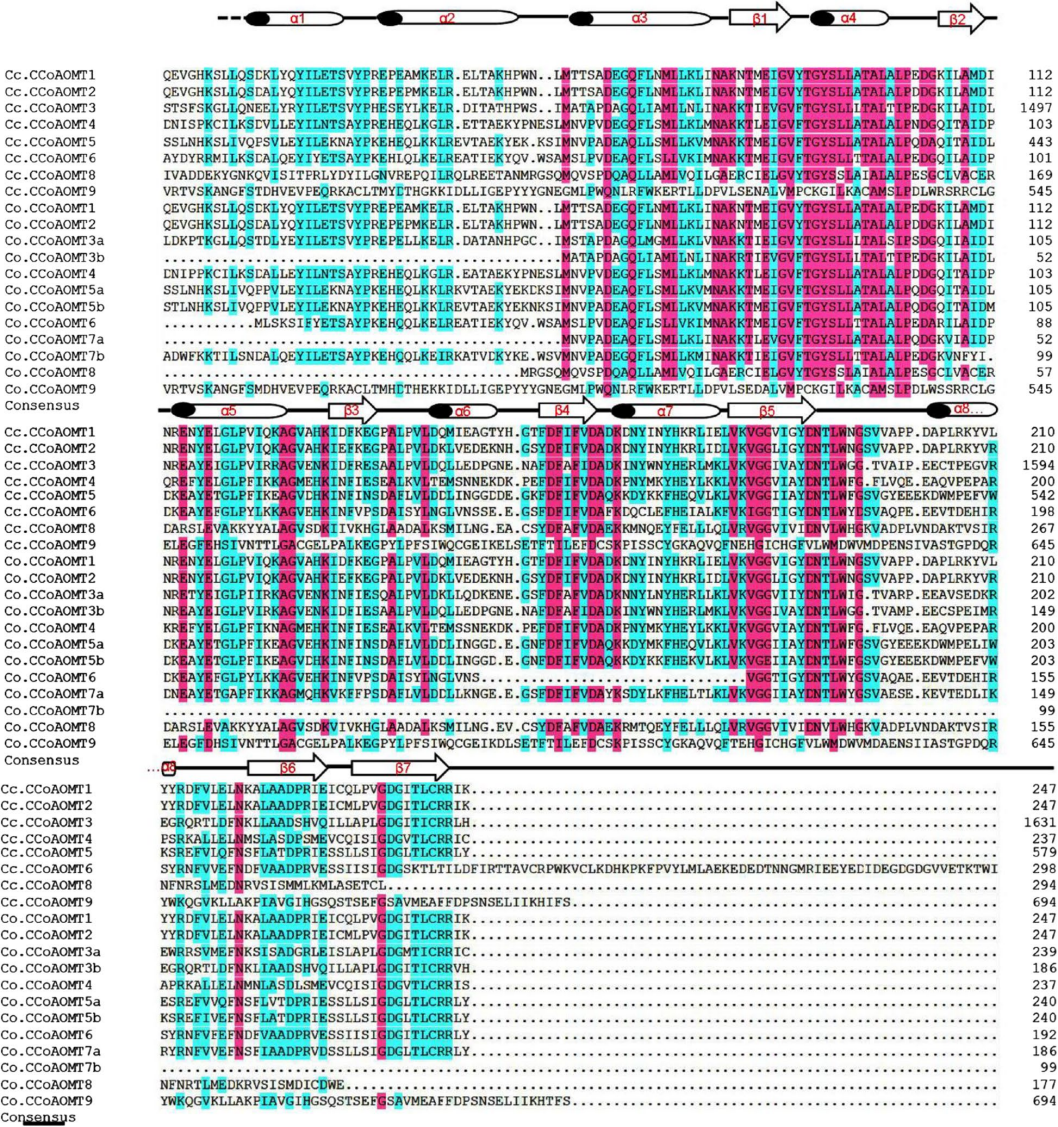
Protein sequence arrangement of the *CCoAOMT* genes in jute. Level of similarity of the CCoAOMT protein sequences were exhibited in dissimilar colors (cyan, cherry red). α-helices depicted as cylinders and β-strands as arrows indicate conserved secondary structural elements

The availability of jute (*C. capsularis* and *C. olitorius*) genome sequences [1] made it possible to pinpoint the members of the *CCoAOMT* gene family in jute. Through a comparative genomics approach, 12 *CCoAOMT* genes were identified in *C. olitorius*, referred to as *Co.CCoAOMT1*–*Co.CCoAOMT9*, and eight orthologous genes were identified in *C. capsularis*, referred to as *Cc.CCoAOMT1*–*Cc.CCoAOMT9* (Table 1). For consistency, *C. capsularis* and *C. olitorius* genes were named according to their phylogenetic relationships. These genes were used as references for primer design. DNA sequences from *CCoAOMTs* were cloned using reverse transcription PCR (Table 2, Additional file 1). These cloned *CCoAOMT* sequences were estimated to include complete open reading frames (ORFs). The size of the CCoAOMT proteins ranged from 99 to 1631 amino acid (aa) residues, with an average length of 365.5 aa. The corresponding molecular mass ranged from 11.36 to 181.62 kDa, with an average molecular weight of 41.03 kDa, and the computed theoretical isoelectric points ranged from 4.61 to 8.93 (Table 3). An analysis of the subtracted protein sequences of *CCoAOMTs* demonstrated that they are highly conserved. All Cc.CCoAOMT and Co.CCoAOMT proteins share a conserved domain which is “AdoMet_MTases” with the exception of Cc.CCoAOMT2 and Co.CCoAOMT2 proteins which share “PLN02589” as their conserved domain (Additional file 2). Six of the *Cc.CCoAOMTs* and eleven of the *Co.CCoAOMTs* shared a conserved domain (AdoMet_MTases) with *A. thaliana* and *Oryza sativa* (Additional file 2). In addition to seven â-strands, eight á-helices and a typical á/â Rossmann fold were discovered in both types of proteins (Fig. 1). Comparative analysis of protein sequences showed that CCoAOMTs exhibited protein sequence similarity, ranging from 20% to 100% (Table 4). Pairwise comparisons among the Co.CCoAOMTs showed that these genes shared protein sequence similarities that ranged from 19.30% to 94.17% with an average of 51.63%, while among the Cc.CCoAOMTs varied from 20.75% to 91.09% with an average of 46.09% (Additional file 3). Using the MEME online tool, a comparison of dissimilarity in protein structure was made to identify the conserved motifs among the jute CCoAOMT proteins. Altogether, 12 distinct motifs were discovered of CCoAOMTs in jute (Additional file 4).

**Table 1:** Basic information of the putative *CCoAOMT* gene family in jute

Gene name	Gene ID	Protein ID	Conserved domain
*Cc.CCoAOMT1*	Cc56659.1	OMO56659.1	AdoMet_MTases
*Cc.CCoAOMT2*	Cc74099.1	OMO74099.1	PLN02589
*Cc.CCoAOMT3*	Cc67343.1	OMO67343.1	AdoMet_MTases
*Cc.CCoAOMT4*	Cc05166.1	OMP05166.1	AdoMet_MTases
*Cc.CCoAOMT5*	Cc60496.1	OMO60496.1	AdoMet_MTases
*Cc.CCoAOMT6*	Cc92789.1	OMO92789.1	DUF295
*Cc.CCoAOMT8*	Cc64145.1	OMO64145.1	AdoMet_MTases
*Cc.CCoAOMT9*	Cc59665.1	OMO59665.1	AdoMet_MTases
*Co.CCoAOMT1*	Co01966.1	OMP01966.1	AdoMet_MTases
*Co.CCoAOMT2*	Co63248.1	OMO63248.1	PLN02589
*Co.CCoAOMT3a*	Co91632.1	OMO91632.1	AdoMet_MTases
*Co.CCoAOMT3b*	Co91633.1	OMO91633.1	AdoMet_MTases
*Co.CCoAOMT4*	Co72425.1	OMO72425.1	AdoMet_MTases
*Co.CCoAOMT5a*	Co88177.1	OMO88177.1	AdoMet_MTases
*Co.CCoAOMT5b*	Co88176.1	OMO88176.1	AdoMet_MTases
*Co.CCoAOMT6*	Co79528.1	OMO79528.1	AdoMet_MTases
*Co.CCoAOMT7a*	Co88174.1	OMO88174.1	AdoMet_MTases
*Co.CCoAOMT7b*	Co88175.1	OMO88175.1	AdoMet_MTases
*Co.CCoAOMT8*	Co82348.1	OMO82348.1	AdoMet_MTases
*Co.CCoAOMT9*	Co52452.1	OMO52452.1	AdoMet_MTases

**Table 2:** Comparison of *CCoAOMT* genes between jute and PCR cloning sequences

Gene name	Jute DNA clone	DNA identity (%)
*Cc.CCoAOMT1*	*Cc.CCoAOMT1*	99%
*Cc.CCoAOMT2*	*Cc.CCoAOMT2*	93%
*Cc.CCoAOMT3*	*Cc.CCoAOMT3*	97%
*Cc.CCoAOMT4*	*Cc.CCoAOMT4*	98%
*Cc.CCoAOMT5*	*Cc.CCoAOMT5*	92%
*Cc.CCoAOMT6*	*Cc.CCoAOMT6*	99%
*Cc.CCoAOMT8*	*Cc.CCoAOMT8*	99%
*Cc.CCoAOMT9*	*Cc.CCoAOMT9*	98%
*Co.CCoAOMT1*	*Co.CCoAOMT1*	99%
*Co.CCoAOMT2*	*Co.CCoAOMT2*	98%
*Co.CCoAOMT3a*	*Co.CCoAOMT3a*	99%
*Co.CCoAOMT3b*	*Co.CCoAOMT3b*	99%
*Co.CCoAOMT4*	*Co.CCoAOMT4*	99%
*Co.CCoAOMT5a*	*Co.CCoAOMT5a*	98%
*Co.CCoAOMT5b*	*Co.CCoAOMT5b*	99%
*Co.CCoAOMT6*	*Co.CCoAOMT6*	98%
*Co.CCoAOMT7a*	*Co.CCoAOMT7a*	99%
*Co.CCoAOMT7b*	*Co.CCoAOMT7b*	99%
*Co.CCoAOMT8*	*Co.CCoAOMT8*	97%
*Co.CCoAOMT9*	*Co.CCoAOMT9*	99%

**Table 3:** Comparison of the characterization of the *CCoAOMT* gene family in jute

Gene name	Protein size (aa)	MW (KDa)	PI	Subcellular	localization
				Signalp/Chlor-op/	Plant-mPloc
				MitoProt	
*Cc.CCoAOMT1*	247	27.83	5.56	N/43.1^*c*^/30.0^*m*^	Chloroplast
*Cc.CCoAOMT2*	247	27.92	5.57	N/43.0^*c*^/21.6^*m*^	Cytoplasm
*Cc.CCoAOMT3*	1631	181.62	5.72	N/47.1^*c*^/65.6^*m*^	Nucleus
*Cc.CCoAOMT4*	237	26.76	5.07	N/43.0^*c*^/4.6^*m*^	Mitochondrion
*Cc.CCoAOMT5*	576	65.08	4.97	N/43.3^*c*^/13.1^*m*^	Chloroplast
*Cc.CCoAOMT6*	400	45.69	4.61	N/42.7^*c*^/11.4^*m*^	Chloroplast
*Cc.CCoAOMT8*	294	32.66	8.93	N/57.6^*c*^/88.6^*m*^	Chloroplast
*Cc.CCoAOMT9*	694	77.72	5.60	N/43.8^*c*^/11.6^*m*^	Chloroplast
*Co.CCoAOMT1*	247	27.83	5.56	N/43.1^*c*^/30.0^*m*^	Chloroplast
*Co.CCoAOMT2*	247	27.92	5.57	N/43.0^*c*^/21.6^*m*^	Cytoplasm
*Co.CCoAOMT3a*	239	26.78	4.91	N/42.8^*c*^/8.4^*m*^	Chloroplast
*Co.CCoAOMT3b*	186	20.54	4.78	N/44.2^*c*^/2.1^*m*^	Plasma membrane
*Co.CCoAOMT4*	237	26.68	4.99	N/42.8^*c*^/4.1^*m*^	Chloroplast
*Co.CCoAOMT5a*	240	27.27	4.86	N/42.9^*c*^/8.6^*m*^	Cytoplasm
*Co.CCoAOMT5b*	240	27.36	4.95	N/42.8^*c*^/8.7^*m*^	Chloroplast
*Co.CCoAOMT6*	192	21.62	4.85	N/43.4^*c*^/6.3^*m*^	Plasma membrane
*Co.CCoAOMT7a*	186	20.76	4.89	N/43.7^*c*^/4.6^*m*^	Chloroplast
*Co.CCoAOMT7b*	99	11.36	5.79	N/42.7^*c*^/4.3^*m*^	Cytoplasm
*Co.CCoAOMT8*	177	19.53	5.07	N/43.4^*c*^/8.9^*m*^	Chloroplast
*Co.CCoAOMT9*	694	77.75	5.50	N/44.0^*c*^/13.6^*m*^	Chloroplast

*Note*: *m* Prospect (%) of marking to mitochondrion, *c* Prospect (%) of marking to chloroplast, *N* Non-secretory protein

**Table 4:** Pairwise comparisons of amino acid sequence among *CCoAOMT* genes in jute

	*Cc.CCo-AOMT1*	*Cc.CCo-AOMT2*	*Cc.CCo-AOMT3*	*Cc.CCoAO-MT4*	*Cc.CCoAO-MT5*	*Cc.CcoAO-MT6*	*Cc.CcoAO-MT8*
*Co.CCoAOMT1*	100%	91%	61%	59%	53%	51%	39%
*Co.CCoAOMT2*	91%	100%	61%	59%	52%	52%	40%
*Co.CCoAOMT3a*	62%	64%	96%	57%	52%	54%	41%
*Co.CCoAOMT3b*	59%	59%	95%	54%	52%	49%	36%
*Co.CCoAOMT4*	58%	60%	52%	95%	60%	59%	40%
*Co.CCoAOMT5a*	52%	52%	52%	62%	93%	62%	35%
*Co.CCoAOMT5b*	54%	53%	50%	62%	94%	61%	36%
*Co.CCoAOMT6*	46%	47%	44%	53%	56%	82%	35%
*Co.CCoAOMT7a*	55%	55%	54%	60%	76%	67%	39%
*Co.CCoAOMT7b*	62%	62%	58%	71%	79%	71%	41%
*Co.CCoAOMT8*	40%	40%	36%	36%	37%	40%	96%
*Co.CCoAOMT9*	24%	25%	20%	28%	29%	22%	24%

Remarkably, two pairs of tandem duplicated genes (*Co.CCoAOMT3a* & *Co.CCoAOMT3b*, *Co.CCoAOMT5a* & *Co.CCoAOMT5b*) were discovered in the *C. olitorius* genome, and were likely originated from the *C. olitorius* as these two tandem duplicated genes of *CCoAOMTs* were not present in *C. capsularis* (Additional file 5). These two pairs of *CCoAOMTs* shared high (71.89% and 94.17%) sequence similarity, suggesting that they were originated from the very recent tandem duplication event (Additional file 5).

**Fig. 2:**
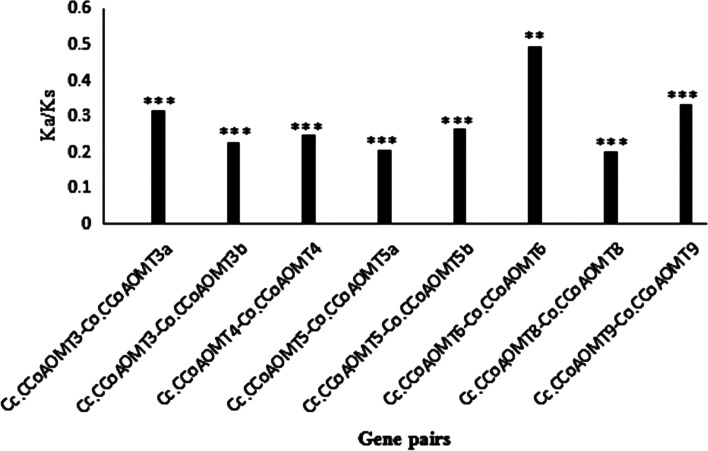
The synonymous (Ks) and non-synonymous (Ka) interchange percentages of 8 CCoAOMT members of jute. For statistical analysis of the ka/ks *p*-value, student's t-test was performed. ** refers *p* value < 0.01 and *** refers *p* value < 0.001 respectively

Analysis of the synonymous to non-synonymous interchange ratio (Ka/Ks) was performed to examine the evolutionary functional hindrance in these jute genes. The results showed there are no non-synonymous sites in gene pairs of *Cc.CCoAOMT1-Co.CCoAOMT1* and *Cc.CCoAOMT2-Co.CCoAOMT2*. Meanwhile, the Ka/Ks ratios of the other six *CCoAOMT* gene pairs investigated were below 0.5, indicating that purifying selection was the principal driver of the evolution of *CCoAOMT* members (Fig. 2, Additional file 6).

### Phylogenetic analysis of the *CCoAOMT* family in 12 different plant species

**Fig. 3:**
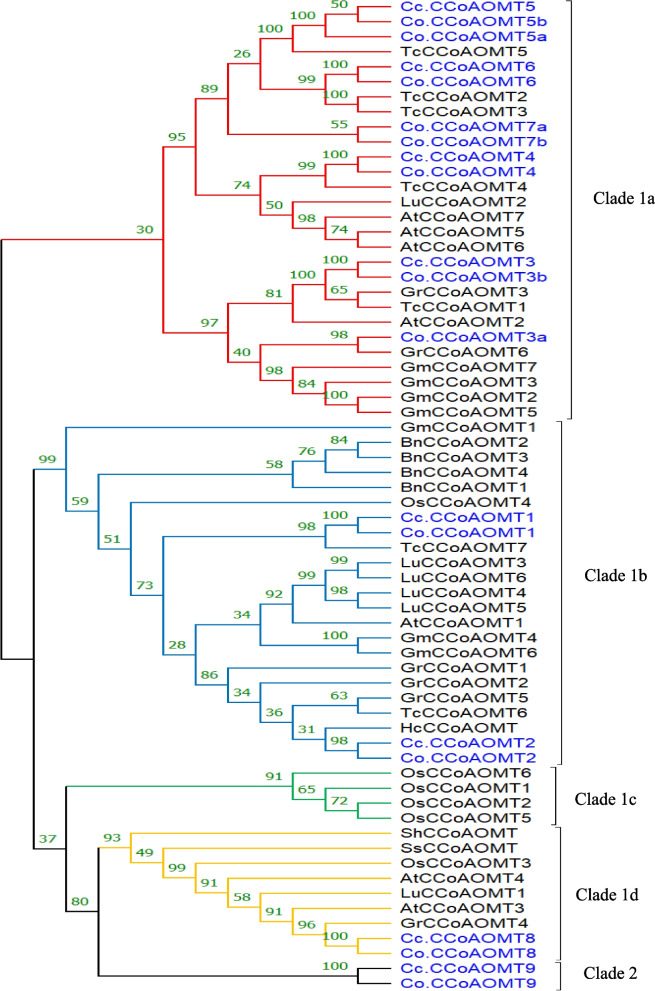
The phylogenetic analysis of CCoAOMTs in twelve plant species. Unrooted phylogenetic tree of CCoAOMT proteins formed using the Neighbor-joining approach with MEGA7 software. Note: Cc.CCoAOMT: *C*.*capsularis*, Co.CCoAOMT: *C*.*olitorius*, *AtCCoAOMT*: *Arabidopsis Thaliana*, *OsCCoAOMT*: *Oryza sativa*, *BnCCoAOMT*: *Boehmeria Nivea*, *GmCCoAOMT*: *Glycine max*, *HcCCoAOMT*: *Hibiscus cannabinus*, *GrCCoAOMT*: *Gossypium Raimondii*, *LuCCoAOMT*: *Linum usitatissimum*, *TcCCoAOMT*: *Theobroma Cacao*, *SsCCoAOMT*: *Synechocystis sp*, *ShCCoAOMT*: *Streptomyces hygroscopicus*, respectively

**Fig. 4:**
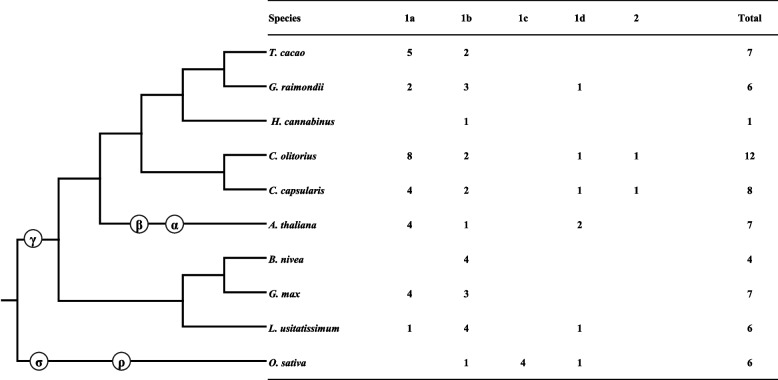
The distribution of *CCoAOMT* gene family members in ten plant species. α, β, γ, σ and ρ represent whole genome duplication events

To broadly assay the evolutionary relationships between the *CCoAOMT* gene family of jute and those of ten plants, we arranged 66 protein sequences from representatives of *A. thaliana*, *Boehmeria nivea*, *Hibiscus cannabinus*, *Glycine max*, *Gossypium raimondii*, *Linum usitatissimum*, *Theobroma cacao, Synechocystis sp, Oryza sativa*, and Streptomyces hygroscopicus as an outgroup, to construct an un-rooted phylogenetic tree using Mega 7.0 (Fig. 3, Additional files 7 and 8). Based on this phylogenetic analysis, CCoAOMTs were divided into two groups, referred to as group 1 (1a, 1b, 1c, 1d) and group 2, consistent with the distribution shown in Fig. 4. Group 1a was the largest, consisting of 28 CCoAOMTs, whereas groups 1b, 1c, 1d and 2 contained 23, 4, 9 and 2 CCoAOMTs*,* respectively. This difference in the number of *CCoAOMT* genes was mainly due to the occurrence of gene gain or loss in the two groups, independent of the level of similarity between the organisms.

Among these two groups, subclade 1c had only Os.CCoAOMT proteins; in contrast, genes in subclades 1a, 1b, 1c, 1d and clade 2 included diverse plant species. The *CCoAOMT* genes could be classified into two groups in the phylogenetic tree, with *Cc.CCoAOMT1*/*2*/*3/4/5/6/8* and *Co.CCoAOMT1*/*2*/*3a*/*3b/4/5a/5b/6/7 s/7b* in one group and the remaining genes in the other group, suggesting that *CCoAOMT*s originated from two ancestral genes. The earliest diverging bacteria (*Streptomyces hygroscopicus*) carried only one *CCoAOMT*, whereas the remaining included 65 *CCoAOMTs* (Figs. 3 and 4). Among the two clades, subclade 1a was found to include the largest number of *CCoAOMTs* (Figs. 3 and 4).

*Co.CCoAOMT3a* was more closely related to other plant *CCoAOMTs* than to its orthologous gene in *C. capsularis*. Finally, *Co.CCoAOMT9* and *Cc.CCoAOMT9* created their own distinct clade beyond the outgroup gene, indicating that they are evolutionarily ancient.

The distribution of *CCoAOMTs* revealed that the increase in the number of *CCoAOMTs*, many of which were species specific, could be attributed to an enormous duplication rate. Altogether, two clades, clade 1 (1a, 1b, 1c, 1d) and 2 included *CCoAOMTs* from both jute and other species, showing that these *CCoAOMTs* were greatly conserved across species during plant evolution. Intriguingly, the distribution of *CCoAOMTs* in the investigated plant species demonstrated that ancient whole-genome duplications (WGDs) did not lead to the expansion of the *CCoAOMT* family (Fig. 4); however, the number of *CCoAOMTs* slowly increased in every group. Phylogenetic analysis also revealed that Cc.CCoAOMTs and Co.CCoAOMTs have very close affinity relationship. However, these genes are more closely related to those of *T. cacao* and *G. raimondii* because they always clustered together with one of them in subclades (1a, 1b and 1d), indicating that *TcCCoAOMTs* and *CrCCoAOMTs* can be utilized as references for jute genes.

We estimated the divergence time among the two clades of *CCoAOMTs* based on the pairwise synonymous substitution rates (Ks) in jute. The median values of pairwise Ks varied from 1.882 to 4.098, corresponding to a divergence time varying from 154.2 to 335.9 Mya, revealing that the *CCoAOMT*s were ancient and divergent (Additional file 9). Furthermore, the divergence time among the paralogous *Co.CCoAOMTs* (5a, 7a, 7b) varied from 54.95 to 389.82 Mya with an average of 140.48 Mya (Additional file 10). These results show that the *Co.CCoAOMTs* are ancient gene family with recent gene duplication events in jute.

### Structural analysis of the *CCoAOMT* genes of jute and other species

**Fig. 5:**
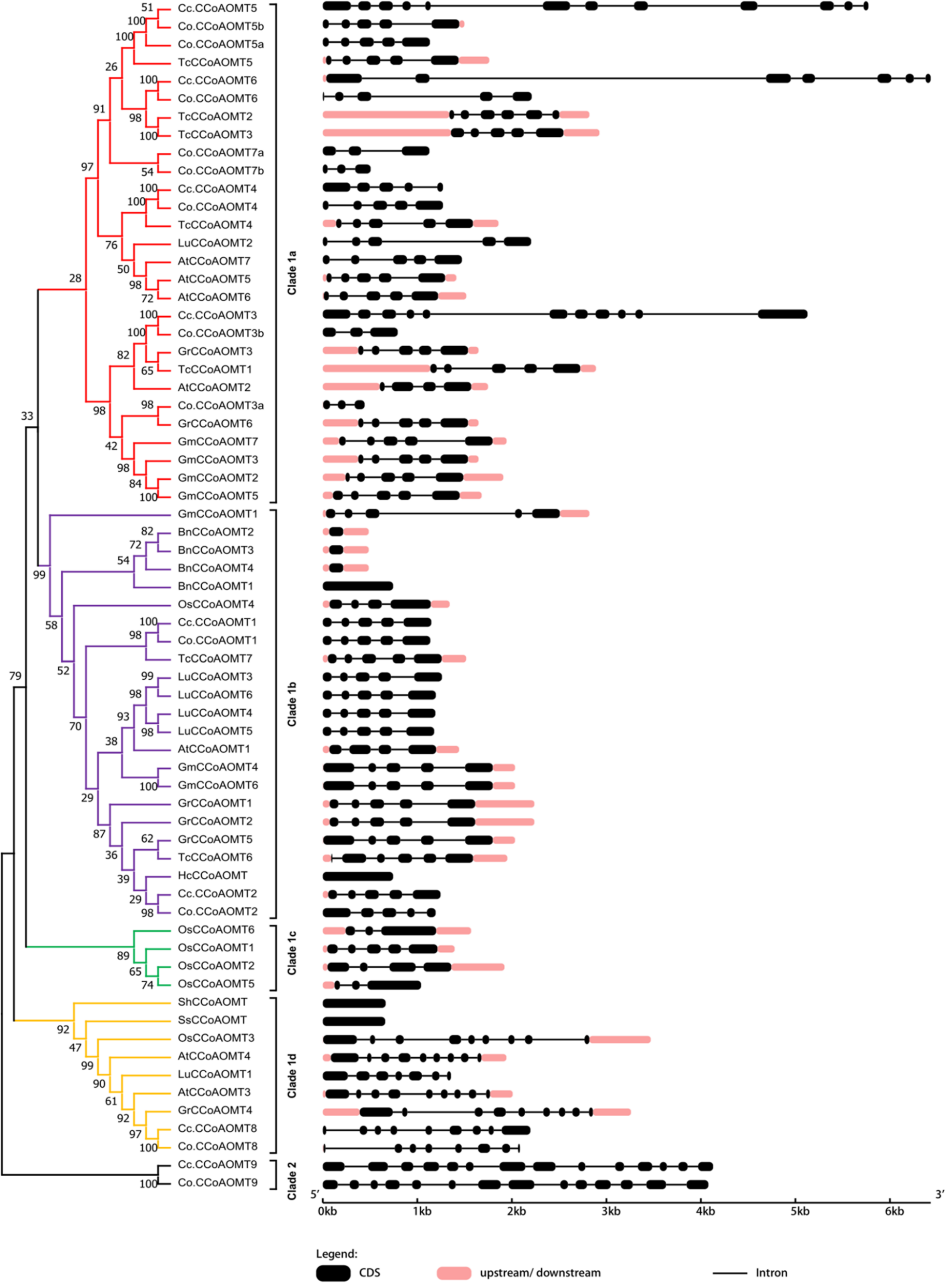
Phylogeny and schematic diagram for exon–intron organization of *CCoAOMT genes* from12 plant species

Based on gene structural analysis, the *CCoAOMT* genes of jute and other species could be divided into two clades (Fig. 5). To examine the structural features and evolution of *CCoAOMTs* in different species, we assayed the structural characteristics and patterns of *CCoAOMTs. CCoAOMT* genes showed a high variability in exon number and size. The exon number of *CCoAOMTs* ranged from 1 to 14 with an average number about 5, and of the 66 analyzed *CCoAOMTs*, 54 *CCoAOMTs* possess a number of exons varying from one to six (Additional files 11 and 12)*,* and their introns are arranged following the GT-AG rule for splicing sites (Fig. 5). Therefore, we theorized that the number of exons of the *CCoAOMT* gene of the last common ancestor (LCA) of angiosperms was between one and six. *CCoAOMT* genes within the same subgroup exhibiting similar exon/intron patterns, particularly in paralogous gene pairs, and most of them possessed a conserved exon/intron pattern in terms of gene length or number of introns.

*Cc.CCoAOMT3*, *Cc.CCoAOMT5*, and *Cc.CCoAOMT*6 have larger frontal introns than their corresponding orthologous genes in *C. olitorius* (Fig. 5). *Co.CCoAOMT3a*/*b* and *Co.CCoAOMT7a*/*b* have three exons, and *Cc.CCoAOMT4* has only five exons, a small number compared to that of the other *CCoAOMT* genes of jute. In subclade 1a, the number of exons of *CCoAOMTs* ranges from three to twelve, while that of *Co.CCoAOMTs*/*Cc.CCoAOMTs* is conserved. *Cc.CCoAOMT1* and *Cc.CCCoAOMT2* showed great similarity in terms of exon/intron number and size with their orthologous genes in *C. olitorius*, consistent with their amino acid sequence similarity; however, they varied in terms of exon/intron patterns (Fig. 5 and Table 4). *Cc.CCoAOMT1*/*2* have one more exon than monocot *CCoAOMTs*, which was likely originated by exonization. In subclade 1b, the number of exons of *CCoAOMTs* ranged from one to five. In subclade 1c, the number of exons of *CCoAOMTs* ranged from three to five, but the gene structure was conserved. *CCoAOMTs* of subclade 1d varied from 1 to 9 exons. In this subclade, *Cc.CCoAOMT8* and *Co.CCoAOMT8* were found to have dissimilar intron sizes; however, they shared a similar gene structure (Fig. 5). These results indicated that *CCoAOMTs* underwent gene restructuring under dissimilar evolutionary dynamics in jute. In clade 2, *CCoAOMTs* had only two exons.

### Chromosomal distribution, gene duplication and collinearity and synteny analysis

**Fig. 6:**
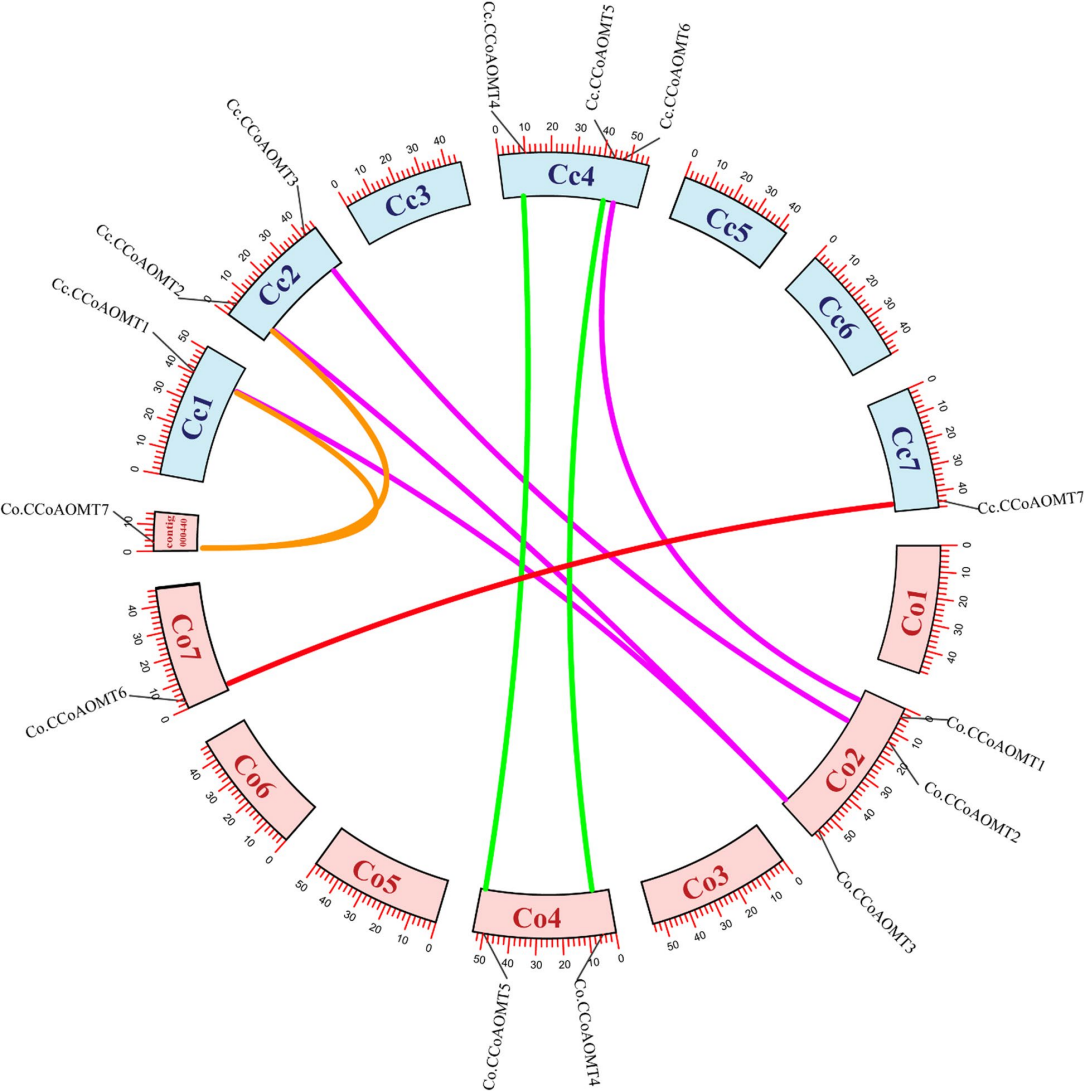
Collinear analysis of *CCoAOMT* gene pairs in jute genome. *CCoAOMT* gene pairs present on duplicated chromosomal segments are connected by different colored lines according to different classes. Col to Co7 (light blue blocks) represent* C*. *olitorius* chromosomes, while Ccl-Cc7 (pink color blocks) represent C. *capsularis* chromosomes

We estimated gene duplication events in the *C. capsularis* and *C. olitorius* genomes by collinearity analysis. A total of 7 collinearity pairs of *Co.CCoAOMT* genes and 7 of *Cc.CCoAOMT* genes were identified by Blastp for all protein sequences and evaluated with MCScanX (Fig. 6). The results showed that 14 genes mapped to jute genome chromosomes, 7 to C. *capsularis* genome, 6 to C. *olitorius* genome and one to contig. The collinearity relationships revealed that over half of the *Co.CCoAOMT* and *Cc.CCoAOMT* collinearity genes were concentrated in Cc2, Cc4, Co2 and Co4 chromosomes, respectively (Fig. 6). Some of the chromosomes (Cc2, Cc4, Co2 and Co4) contained three or two *CCoAOMT* genes, while some others (Cc1, Cc7 and Co7) contained only one gene. There were no *CCoAOMT* genes identified on chromosomes Cc3, Cc5, Cc6 in *C. capsularis* and Co1, Co3, Co5, Co6 in *C. olitorius* (Fig. 6). The absence and uneven distribution of *CCoAOMT* genes on some *C. capsularis* and *C. olitorius* genome chromosomes indicate that gene loss may have occurred during evolution but an incomplete genome assembly could also be a factor.

Additionally, the analysis of genetic evolution of the *CCoAOMT* members was carried out by the synteny analysis among *C. capsularis* and *C. olitorius* with the *CCoAOMT* genes of *A. thaliana*, and *Oryza sativa* (Additional file 13). Most of *Cc.CCoAOMTs* and *Co.CCoAOMTs* are paired with *A. thaliana*, producing a total of 5 collinear pairs. However, it was found that *Cc.CCoAOMT3* was paired with two genes from *A. thaliana*, while *Cc.CCoAOMT9* was paired with an orthologous gene from *A. thaliana* and *Oryza sativa* (Additional file 13). Therefore, it was observed that the strongest collinearity relationships are between dicot plants.

### Gene expression and function of *CCoAOMTs* in jute

**Fig. 7:**
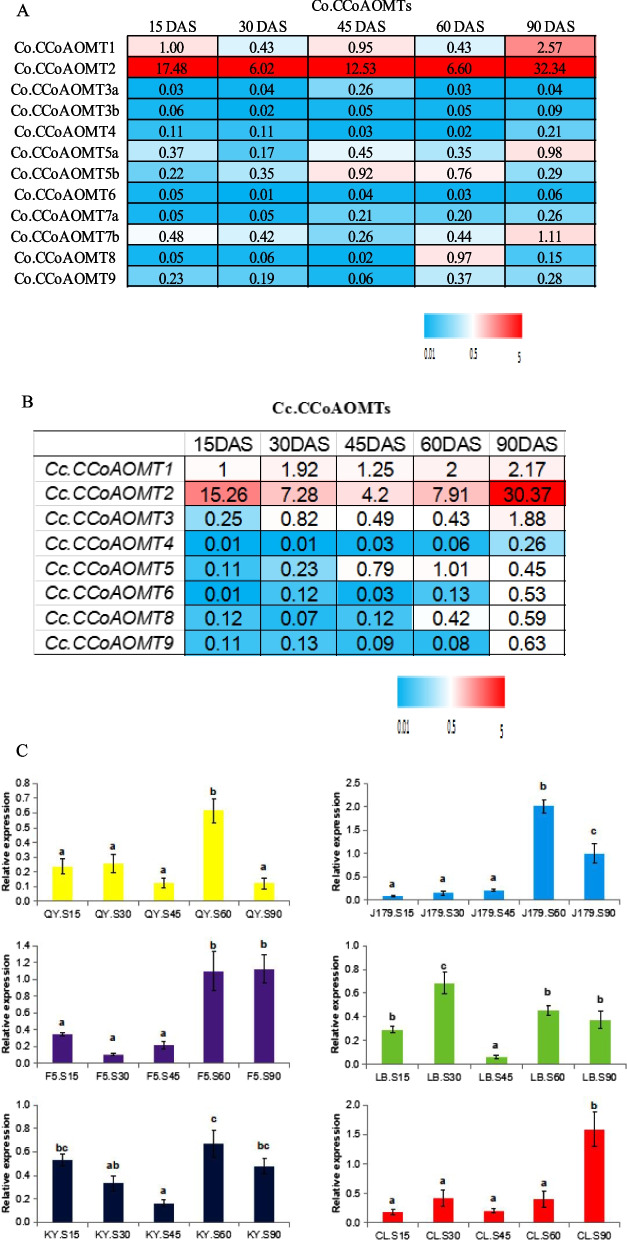
Transcript levels of *CCoAOMT* genes under different growth stages and varieties of jute. **A** The transcript levels of *CCoAOMT* members in the stem of *C. olitorius* at five dissimilar developmental stages (15, 30, 45, 60 and 90 DAS) under F5 variety. **B** The expression patterns of *CCoAOMT* genes in C. *capsularis* under five different developmental stages (15, 30, 45, 60 and 90 DAS) in J179 variety. DAS refers days after sowing. **C** Comparison of expression patterns of *CCoAOMT2* in two jute species (*C*. *olitorius* and *C*. *capsularis*) under different developmental stages and varieties. Note: SDs and mean values were acquired from three biological replicates. The letter S refers to stage. Turkey test was used for statistical analysis, and different letters expressed significant differences between developmental stages (*p* < 0.05)

Detailed analysis of gene expression can provide an initial reference for assessing the potential function of genes in plants. In this study, all *Cc.CCoAOMT* and *Co.CCoAOMT* expression analysis was conducted using stem samples. The analysis of the result showed that *Cc.CCoAOMT1*/*2* and *Co.CCoAOMT1*/*2* possessed high transcript levels at all developmental stages (Fig. 7), suggesting that these two genes are the principal members of the *CCoAOMT* gene family. Conversely, *Cc.CCoAOMT6* and *Co.CCoAOMT3a*/*3b*/*4*/*6* showed low transcript levels at all developmental stages and in all examined stem tissues (Fig. 7A, B). Also, the expression of *Cc.CCoAOMT4* was almost undetectable at all developmental stages and in all tissues investigated. Finally, *Cc.CCoAOMT3*/*5*/*8*/*9* and *Co.CCoAOMT5a*/*5b*/*7a*/*7b*/*8*/*9* exhibited differential gene transcript profiles in the two jute species at different developmental stages.

### *CCoAOMT* expression levels at different developmental stages

*CCoAOMT* genes could be categorized into three types according to their expression patterns: period-specific expression, constitutive expression, and low expression. Among the 12 assayed *CCoAOMT* genes from *C. olitorius*, four genes (*Co.CCoAOMT3a*, *Co.CCoAOMT3b*, *Co.CCoAOMT4*, and *Co.CCoAOMT6*) showed undetectable or very low transcript levels across the different growth stages. In contrast to other genes, *Co.CCoAOMT1* showed differential gene expression levels in these five developmental stages and displayed the highest transcript levels at 90 DAS (Fig. 7A). *Co.CCoAOMT2* was the most highly expressed among the members of *C. olitorius CCoAOMT* family at different developmental stages (F5 variety), and exhibited a constitutive transcript level (Fig. 7A). *Co.CCoAOMT2* displayed higher expression levels at 60 and 90 DAS than at 15, 30, or 45 DAS (Fig. 7C), and higher expression in F5 than in the other two varieties (CL and KY) at 60 DAS (Additional file 14). Additionally, this gene exhibited the highest transcript levels in F5 and CL at 90 DAS (Additional file 14). Overall, during the early developmental stages from 15 to 45 DAS, the *CCoAOMT*2 expression levels were relatively low and had no significant difference among each stage except LB and KY (Fig. 7C). Furthermore, the *CCoAOMT2* expression levels of most cultivars reached highest expression level during maturing stages at 60 DAS and 90 DAS except LB, which reached the highest at 30 DAS (Fig. 7C). At the seedling stage, *Co.CCoAOMT7a* showed low transcript levels; thus, *Co.CCoAOMT7a* probably plays a role in the premature development of jute. In contrast to that of *Co.CCoAOMT7a*, the expression of *Co.CCoAOMT7b* was abundant at all five developmental stages; however, this gene displayed greater transcript levels in mature stems than in premature ones (Fig. 7A). Both *Co.CCoAOMT8* and *Co.CCoAOMT9* were found to display greater transcript levels at 60 and 90 DAS than at 45 DAS. Finally, *Co.CCoAOMT5a*, *Co.CCoAOMT5b*, *Co.CCoAOMT7b*, *Co.CCoAOMT8*, and *Co.CCoAOMT9* exhibited differential expression patterns across the five developmental stages (Fig. 7A).

The eight *Cc.CCoAOMTs* of *C. capsularis* generally followed a similar expression pattern to that of their orthologous genes in *C. olitorius*. Only a slight difference was observed in the expression of *Cc.CCoAOMT8*/*9*, which was lower at seedling stages (15 and 30 DAS) and greater at the mature stage (90 DAS) than that of their orthologs (Fig. 7B). In contrast to *Co.CCoAOMT3a*, *Cc.CCoAOMT3* displayed high expression levels during the mature stage (Fig. 7B). Similarly, *Cc.CCoAOMT1*/*2* exhibited higher expression levels than those of their orthologous genes in *C. olitorius* (Fig. 7B). The abundance of *Co.CCoAOMTs* and *Cc.CCoAOMTs* were compared for their relative transcript patterns at 15, 30, 45, 60, and 90 DAS by normalizing with the lowest expressed gene and presented as fold change in expression (Additional file 15).

To identify the expression levels of *Co.CCoAOMTs* and *Cc.CCoAOMTs* at different parts (top, middle and bottom) of the *C. capsularis* and *C. olitorius* stem tissues, a comparison of their transcript patterns was carried out. For *Co.CCoAOMTs*, the results exhibited that these genes were deferentially expressed at the top, middle and bottom tissues, with an average expression of these genes being higher in the middle tissues and mild in the top or the bottom, suggesting a gradient of *CCoAOMT* activity in the *C. olitorius*. Similarly, the expression levels of *Cc.CCoAOMTs* showed differential expression, with an average transcript being greater in the bottom tissues and moderate in the top or the middle (Additional file 16).

To comprehend the overall function of *CCoAOMTs *during different developmental stages of the jute stem, the integrated transcript levels of all *CCoAOMTs* were calculated. This analysis revealed a 0.5-fold decrease in *CCoAOMT* gene expression between 15 and 30 DAS, a 0.12-fold increase between 30 and 45 DAS, a 0.02-fold decrease between 45 and 60 DAS, and a 3.4-fold increase between 60 and 90 DAS (Additional file 17). Overall, the transcription of *CCoAOMTs* was enhanced throughout jute stem growth, consistent with their major role in lignin biosynthesis. In particular, *CCoAOMT2* was the most abundantly expressed *CCoAOMT* across all developmental stages and species. Therefore, this gene was selected for further analysis, and its expression patterns were compared for six different varieties and phases (Fig. 7C). The results revealed that the expression of this gene was enhanced gradually in parallel with the growth of the plant and reached its peak at the mature stages (60 and 90 DAS) (Fig. 7C).

### Analysis of lignin content in different varieties at various developmental stages

**Fig. 8:**
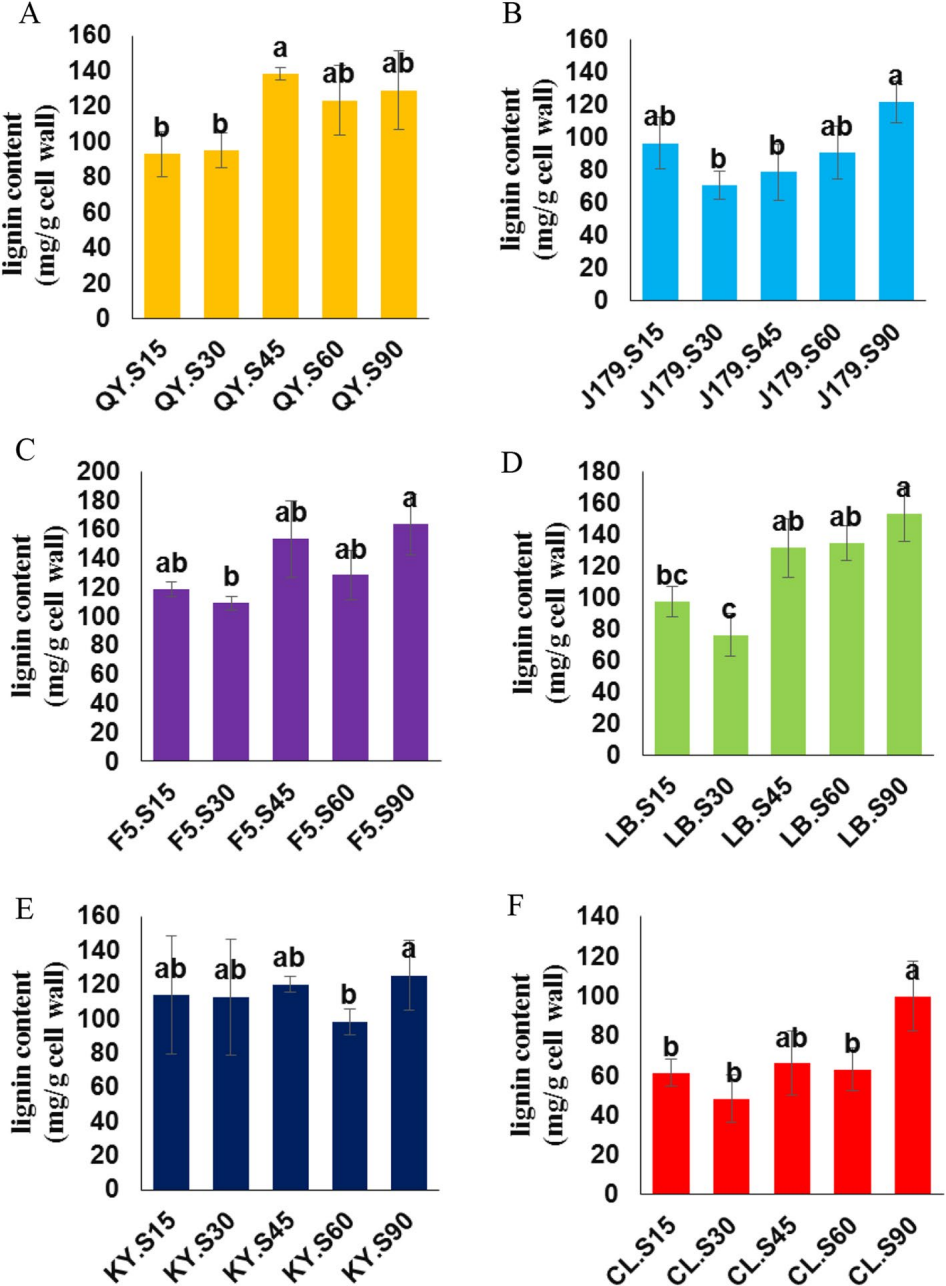
Comparison of lignin content under dissimilar growth stages and varieties in jute. **A** QY variety under different stages. **B** J179 variety under different stages. **C** F5 variety under different stages. **D** LB variety under different stages. **E** KY variety under different stages. **F** CL variety under different stages. Note: SDs and mean values were acquired from three biological and five technical replicates. One-way ANOVA with Fisher’s test was used for statistical analysis, and different letters expressed significant differences between developmental stages (*p* < 0.01). The letter S refers to stage

The measurement of lignin content using the acetyl bromide method revealed an overall increase in the lignin content of jute during fiber development (Fig. 8). The lignin content of J179, QY, LB, F5, and CL at the mature stage (90 DAS) increased by 20.7%, 28%, 36.3%, 27.5%, and 38.6%, respectively, compared with that of the premature stage (15 DAS) (Fig. 8A, B, C, E, F). This drastic increase in lignin content was observed during the transition from the vegetative to the reproductive growth phase, and was mainly ascribed to the lignification triggered by *CCoAOMTs*. The lignin content of J179, LB, and CL decreased at 30 DAS, but then increased steadily in parallel with the growth of the plant (Fig. 8B, C, F). The lignin content of QY increased steadily along plant development, peaking at stage 45 DAS (Fig. 8A), whereas that of F5 fluctuated during plant growth (Fig. 8C). The lignin content of KY did not show any substantial increment during seedling and vegetative phases (15, 30 and 45 DAS) but suddenly dropped at stage 60 and finally increased at maturing stage (90 DAS) (Fig. 8E). Furthermore, the correlation coefficient between lignin content and *CCoAOMT2* gene expression level of each cultivar was inconsistent, both positive and negative correlations were found. Among which, CL variety showed the highest correlation coefficient (0.879), indicating a positive correlation between the lignin content and the *CCoAOMT2* gene expression level in this cultivar (Table 5, Additional file 18). This result indicates that the down-regulation of *CCoAOMTs* may reduce the lignin content of jute (CL variety), thereby enhancing fiber quality.

**Table 5:** Correlation coefficient between lignin content and *CCoAOMT2* expression level

Jute cultivar	Correlation coefficient
J179	0.3514
QY	-0.0945
LB	-0.5016
F5	0.4742
KY	-0.1725
CL	0.8791

*Note*: Pearson’s R correlation coefficient test was used for statistical analysis

### Expression of *CCoAOMTs* under PEG or NaCl treatment

**Fig. 9:**
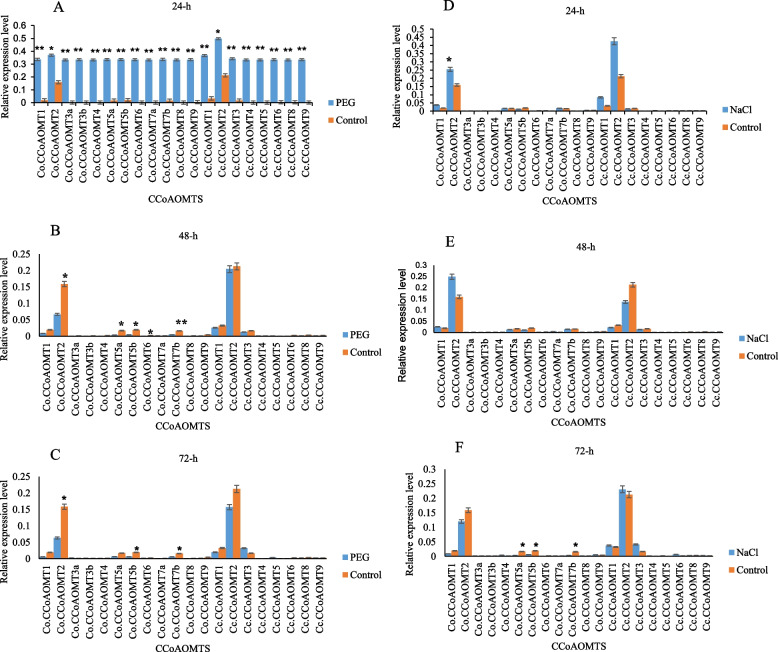
RT-qPCR expression patterns of *CCoAOMT* genes under drought and salt stress. The expression patterns were normalized to 24, 48 and 72 h (drought and salt stress treatments), respectively. A, B and C are expression patterns of *CCoAOMT* genes under PEG treatment; and D, E, F are expression levels of *CCoAOMT* genes under NaCl treatment. The asterisks indicate at most significantly difference (**p < 0.001, *p < 0.01)

The expression of *Co.CCoAOMTs* and *Cc.CCoAOMTs* was examined under NaCl and PEG treatments at various time points (Fig. 9). At 24 h after PEG treatment, the expression of *CCoAOMTs* was highly up-regulated compared to that of control plants (Fig. 9A). In particular, *Cc.CCoAOMT2* was induced by PEG stress, upon which it exhibited higher up-regulation greater than that of the control. The expression of *Co.CCoAOMTs* decreased during the second stage (48 h) of drought stress, whereas the transcript levels of *Cc.CCoAOMTs* were similar to those of control plants (Fig. 9B). The abundance of *Co.CCoAOMT* and *Cc.CCoAOMT* transcripts was substantially reduced at later stages (72 h) (Fig. 9C). This comparative analysis of *CCoAOMT* expression under PEG treatment showed that some *C. olitorius* and *C. capsularis* orthologous genes are either drought repressible or drought inducible in a similar way, indicating that these two species might share common transcriptional responses to drought stress. However, a small number of *C. olitorius* orthologs showed dissimilar responses to drought stress, suggesting the existence of species-specific drought responses.

Under salt stress, the expression of eight genes (*Co.CCoAOMT1*/*2*/*5a*/*5b*/*7b* and *Cc.CCoAOMT1*/*2*/*3*) was greatly induced (Fig. 9D). Nonetheless, more than half of all genes were repressed at 24 h (Fig. 9D). After 48 h from NaCl treatment, the expression levels of five *Co.CCoAOMT* genes (*Co.CCoAOMT1*/*2*/*5a*/*5b*/*7b*) were differentially expressed, whereas their orthologs (*Cc.CCoAOMT1*/*2*/*3*) were down-regulated (Fig. 9E). However, these five *Co.CCoAOMT* genes were down-regulated at 72 h (Fig. 9F). *Co.CCoAOMT1*/*2*/*5a*/*5b*/*7b* were differentially expressed, whereas their orthologues genes (*Cc.CCoAOMT1/2/3*) were down-regulated (Fig. 9E) in response to at least one treatment, and most of them could be induced by more than one stress treatment; this revealed that these genes are involved in the crosstalk between distinct signal transduction pathways in response to abiotic stresses. For example, *Co.CCoAOMT2* was considerably down-regulated by PEG (48 h), while being up-regulated by NaCl treatment (48 h). Relatively greater *Cc.CCoAOMT* transcript levels were observed under drought and salt stress-tolerant cultivar J179, compared to those of the sensitive cultivar F5, confirming the essential role of these genes in the response to drought and salt stresses in jute (Fig. 9). Therefore, our study revealed that *Co.CCoAOMTs* from F5 and *Cc.CCoAOMT* from J179 are greatly induced by drought and salt stress and may play a crucial role in resistance to abiotic stress.

## Discussion

### Evolution of the *CCoAOMT* gene family in jute

Recent studies have investigated and examined the characteristics, evolution, expression, and function of some *CCoAOMT* genes in plants such as *A. thaliana* [31], citrus [32], sorghum [30], and maize [33, 34]. However, very little is known about *CCoAOMT* gene family in jute. In this study, 12 *CCoAOMT* genes from *C. olitorius* and eight *CCoAOMTs* from *C. capsularis* were identified, a higher number than in *A. thaliana* (seven *CCoAOMTs*), *O. sativa* (six *CCoAOMTs*), *G. raimondii* (six *CCoAOMTs*), and *T. cacao* (seven *CCoAOMTs*), suggesting the evolutionary expansion of *CCoAOMT* genes in jute.

Gene duplication events, mainly consisting of segmental and tandem duplications, have been suggested to be the principal driving force for the expansion and evolution of gene families [35]. The model plant *A. thaliana* is known to have undergone several whole genome duplication (WGD) events, resulting in the large-scale expansion of gene families in its genome [36]. The results of this study indicate that the mechanisms underlying *CCoAOMT* gene expansion may differ across species. WGDs are considered to be the typical driving force for the evolution of angiosperms, the latest evolved and most successful lineage of land plants [37–39]. Previous studies have discovered that the genome of pineapple has undergone a less ancient WGD (ñ) event than the other sequenced grass genomes. These currently accessible WGD data, along with the genomes of 10 plant species depicting major WGD events in angiosperms, facilitated the study of *CCoAOMT* gene evolution in angiosperms. New gene functions can be gained through four processes of gene evolution: chromosome insertion, duplication, transposition, and tandem duplication [40–43]. Since jute has only undergone a γ WGD, its gene duplication might be attributed to tandem duplication. It is worth noting that *C. olitorius* and *C. capsularis* have a different number of *CCoAOMT* genes (eight and four, respectively) belonging to group 1a, and this could depend on a recent gene duplication occurred after their speciation.

Based on the phylogenetic analysis of *CCoAOMT* genes from 12 plant species, the *CCoAOMTs* considered in this study could be divided into two clades. Clade 1 and 2 including *CCoAOMT* genes from both *C. capsularis* and *C. olitorius*. In particular, the *Co.CCoAOMT7a*/*b* genes (absent in *C. capsularis*) appeared to be the result of a recent duplication event occurred after the speciation of *C. capsularis* and *C. olitorius*. These two genes, detected only in *C. olitorius*, might have been lost by *C. capsularis* during evolution. Indeed, gene loss could occur due to the accumulation of harmful mutations, which is especially frequent soon after polyploidy events [44]. Phylogenetic analysis also indicated that the *CCoAOMTs* of jute have a closer affinity to *T. cacao* and *G. raimondii CCoAOMTs* than to those of the other investigated plant species, providing clear evidence that *C. capsularis* and *C. olitorius* might have a more recent common ancestor with *T. cacao* and *G. raimondii* than with other plant species. Consistent with the observed phylogenetic relationships, clade 2 *CCoAOMTs* belonged to the oldest branch of the *CCoAOMT* gene family. Therefore, *CCoAOMTs* in this group were hypothesized to have undergone more intron gain/loss events during their lengthy evolutionary process in line with the introns-early theory [45, 46]. Consequently, we presumed that the evolution of the gene structures of clade 2 *CCoAOMTs* was mainly due to exon splitting.

Exon–intron structural differences between orthologous genes have been shown to be generated by three main mechanisms: exon/intron gain/loss, exonization/pseudoexonization, and insertion/deletion [47]. Comparative analyses of the gene structures of *CCoAOMTs* made it possible to evaluate the structural evolution of *CCoAOMT* genes in jute. The *CCoAOMT* genes of jute displayed great variability in the number of exons. Compared with the variable structures of the *CCoAOMT* genes from jute, the sequences of the corresponding proteins were more conserved. Thus, gene structural evolution after the divergence of these plant species did not cause significant differences in the coding region. Therefore, *CCoAOMT* structural variants mainly evolved through intron gain/loss and insertion/deletion, but not through exonization/pseudoexonization. According to their phylogenetic distribution, the evolution of *CCoAOMT* genes in jute travelled along significantly distinct paths over evolutionary history. In addition, the fact that the majority (54.55%) of *CCoAOMTs* have five exons led us to infer a basic gene model containing five exons and four introns for the ancestral *CCoAOMT* genes, from which all observed gene structures could develop by insertion and/or loss of introns. In comparison with the *CCoAOMT* gene structure and exon counts of Physcomitrella patens, Selaginella and Plagipchasma, the *CCoAOMT* gene structure and exon number from the two jute species and the other eight plant species analyzed in this study are more diverse in exon number, size and pattern [30, 48]. The exon number of *CCoAOMT* genes from Physcomitrella patens, Selaginella and Plagipchasma range from three to seven while the exon number of CCoAOMT genes from *C. capsularis, C. olitorius, Oryza sativa, A. thaliana, Boehmeria nivea, Glycine max, Hibiscus cannabinus, Gossypium raimondii, Linum usitatissimum* and *Theobroma cacao* varied from one to fourteen respectively.

The study of variations in *CCoAOMT* gene structures is key to unravel the genome dosage of the two jute species, and further investigation may provide the foundation for understanding the molecular basis of jute genetics.

The orthologous *CCoAOMT* gene pairs of *C. capsularis* and *C. olitorius* exhibited high identity (82% ~ 100%). The structural features of some *Co.CCoAOMTs* corresponded to those of the respective *Cc.CCoAOMTs*, demonstrating that the functions of these genes remained highly conserved during evolution. Moreover, Cc.CCoAOMT6/Co.CCoAOMT6 had the lowest protein sequence similarity (82%) among the orthologous pairs. The pI values of CCoAOMTs of jute varied from 4.61 (Cc.CCoAOMT6) to 8.93 (Cc.CCoAOMT8), with majority of the members (11) showing pI values higher than 5 (Table 3). The pI values of the remaining nine genes were lower than 5, indicating the independence between pI divergence and gene evolution.

The Ka/Ks assay showed that most of the homologous *CCoAOMT* genes from jute have experienced purifying selection, since most *Co.CCoAOMTs* exhibited very high similarity in gene sequences and highly conserved functions to their orthologs from *C. capsularis*. This assay also revealed that *CCoAOMT4* had a lower selective constraint than the other *CCoAOMTs*. Furthermore, *CCoAOMT4* was distinct from the other *CCoAOMTs* in terms of protein size and showed the lowest gene expression levels among all *CCoAOMTs*. Thus, the evolution of *CCoAOMT4* could have depended on its functional specialization in jute, given the long-term divergence of *CCoAOMT4* from the LCA with respect to the other *CCoAOMTs*. In summary, the study of the evolutionary relationships and gene structures of *CCoAOMTs* is a vital step towards the comprehension of their potential functions in jute.

### Expression of *CCoAOMT* genes at different growth stages

Gene transcript profiles are highly correlated with gene function in plants [49]. Previous studies on *CCoAOMT* genes indicated that they may play a role in lignin biosynthesis, disease, and abiotic stress resistance in some plant species [40–53]. In the present study, the expression levels of *CCoAOMTs* were examined using stem tissues at five distinct stages and in different varieties of jute. Among group 1b genes, *CCoAOMT1* showed greater expression at the mature stage (90 DAS) than at the premature stage (15 DAS) in *C. olitorius* than in *C. capsularis*. *CCoAOMT2* was the most highly expressed gene among all *CCoAOMTs*, and its transcript levels peaked at the last stage (90 DAS) (F5, CL varieties), suggesting that this gene is the predominant gene involved in lignin biosynthesis in jute. The *CCoAOMT2* showed different expression levels in the six cultivars at each developmental stage. *Cc.CCoAOMT2* of J179 had relatively lower transcript level at 15 DAS and 30 DAS, while the one in KY and LB varieties had higher expression level at the same stages. At 45 DAS, *Cc.CCoAOMT2* of LB variety expressed relatively low transcription level and showed significant difference with other cultivars. *Cc.CCoAOMT2* expression level of J179 reached highest at 60 DAS, in comparison with other cultivars at the same stage. Moreover, at 90 DAS, QY, LB and KY showed lower transcript levels, in contrast to CL, F5 and J179 had higher transcript level at the same stage. Furthermore, the *Cc.CCoAOMT2* expression levels of QY were relatively low at all stages in comparison with other cultivars. This gene showed higher expression levels in *C. olitorius* than in C. *capsularis*.

Among group 1a genes, *Co.CCoAOMT3a* and *Co.CCoAOMT3b* showed very low transcript levels at all growth stages, while their orthologous *Cc.CCoAOMT3* exhibited greater transcript levels at all five developmental stages, which peaked at 90 DAS. This led us to hypothesize that *CCoAOMT3* may be functionally divergent in the two species.

*Co.CCoAOMT4* and *Cc.CCoAOMT4* were undetectable throughout the developmental stages and in all varieties. *CCoAOMT4* was presumed to have originated after the divergence of *C. olitorius* and *C. capsularis*, in accordince with the results of the phylogenetic analysis described before, supporting the hypothesis that the *CCoAOMT4* lineage is functionally redundant in malvids. Additionally, the lack of an orthologous gene to *Co.CCoAOMT7* in *C. capsularis* was considered to be due to a gene deletion event occurred after the divergence of *C. olitorius* and *C. capsularis. Co.CCoAOMT5b* was highly expressed at the maturing (45 DAS) and mature stages (60 DAS), and its ortholog *Cc.CCoAOMT5* exhibited similar expression levels at the mature stage (60 DAS). *CCoAOMT6* showed very low transcript at all developmental stages and in all investigated tissues, possibly as a result of gene functional redundancy due to the recent ρ WGD. *Co.CCoAOMT7a*/*b* are recently duplicated genes that showed different expression patterns in the examined tissues and developmental stages. The expression of both *Co.CCoAOMT7a* and *Co.CCoAOMT7b* peaked at 90 DAS. Furthermore, the expression of *Co.CCoAOMT7s* dramatically increased from the maturing to the mature phase, suggesting that these genes play a significant role in lignin biosynthesis.

Among group 1d genes, *CCoAOMT8* showed increased expression at the maturation stage (60 DAS) and had higher transcript levels in *C. olitorius* than in *C. capsularis* at the same stage. This gene also exhibited a similar expression pattern in different tissues of the stem in both jute species. Among group 2, the expression of *CCoAOMT9* peaked at the maturation phase and it was lower in *C. olitorius* than in *C. capsularis*, whereas it was undetectable in both species at the premature stage. Overall, gene expression analysis revealed that orthologous genes had similar expression patterns in both species.

In the present study, *CCoAOMT1* and *CCoAOMT2* were found to be constitutively expressed in the whole stem of both species and across all growth stages, indicating their essential role in lignin biosynthesis in jute. Six genes (*Co.CCoAOMT3a*, *Co.CCoAOMT3b*, *Co.CCoAOMT4*, *Co.CCoAOMT6*, *Cc.CCoAOMT4*, and *Cc.CCoAOMT6*) exhibited very low or undetectable transcript levels in all the investigated tissues of the two species and at all growth stages, indicating that these genes may play non-essential roles in lignin biosynthesis in jute.

### Relationship between lignin content and *CCoAOMT* gene expression

Despite being one of the most important fiber sources, jute has relatively high lignin content [1]. Nevertheless, little information is available on lignin biosynthesis in jute. In this study, the expression levels of the lignin-related *CCoAOMT* genes and lignin accumulation patterns in jute were thoroughly examined for the first time. The results showed an overall increase in lignin content during the development (from 30 DAS) of three jute varieties (CL, J179, and LB), whereas the lignin content of the other two varieties (F5, and KY) fluctuated across the five developmental phases. The lignin content of QY enhanced steadily during plant development, peaking at stage 45. A significant increase in lignin content was observed at 90 DAS compared to that at 45 DAS (J179, LB, F5 and CL varieties). Additionally, the analysis of *CCoAOMT* activity revealed greater expression levels at 90 DAS, perfectly correlating with the rapid increase in lignin content occurring at the same stage. The integrated transcript level of all *CCoAOMT* genes was greatest at 90 DAS, further supporting the correlation between *CCoAOMT* gene expression and lignin accumulation. Furthermore, the correlation of lignin content and *CCoAOMT2* expression level was inconsistent among the six jute varieties investigated. A positive correlation between lignin content and *CCoAOMT* expression was observed under CL varieties (Table 5). The production of transgenic jute plants with diverse lignin structures and the least damage from other cell wall constituents could be a useful strategy for examining the impact of lignin properties on fiber quality in the future.

### Analysis of the expression of *CCoAOMTs* under abiotic stresses

Profiling gene expression under many abiotic stresses is vital for a mechanistic understanding of stress resistance in plants. Lignin biosynthesis via the phenylpropanoid pathway is associated to a complex regulatory network, in which secondary metabolites synthesized by various interacting enzymes perform essential functions in stress responses [54]. *CCoAOMT* was first reported to be instantly up-regulated by fungal elicitors in carrot and parsley cell cultures [55]. Subsequent studies indicated that the corresponding enzyme may play a crucial role in tolerance to stresses such as drought, cold, wounding, salt, and phytohormones [56–59]. Even though there is little information on the mechanisms by which drought affects lignin biosynthesis, several studies have demonstrated that *CCoAOMTs* can be considerably or slightly up-regulated by drought at the protein and mRNA levels in the roots of *A. thaliana* [60] and in the leaves of wild watermelon [61], soybean [62], peanut [63], and *H. cannabinus* [57].

Understanding how stressors impact the transcription of *Co.CCoAOMTs* and *Cc.CCoAOMTs* may prompt the development of novel methods for genetic improvement of jute for its cultivation in marginal areas. Our study showed that *Co.CCoAOMTs* and *Cc.CCoAOMTs* were substantially up-regulated at 24 h after exposure to drought stress. This result was consistent with those of Liu et al. [28], who discovered the up-regulation of *CCoAOMTs* in both stems and leaves of switchgrass during later time points (24 ~ 48 h) of drought stress. The expression of *Co.CCoAOMT* genes was down-regulated in dehydrated stem tissues, whereas the transcript levels of *Cc.CCoAOMTs* were unchanged with respect to those in untreated controls at 48 h. In contrast, the transcript levels of both *Co.CCoAOMTs* and *Cc.CCoAOMTs* decreased at 72 h after drought stress.

The lignin content and gene transcript levels might depend on the plant tissue. For instance, the slower growth at the bottom of maize roots in comparison to the top was associated with greater up-regulation of cinnamoyl-CoA reductases (CAD) 1 and 2, and increased deposition of lignin [24]. Interestingly, we discovered that the expression of *CCoAOMTs* in the stems of jute after PEG treatment was substantially greater at 24 h (approximately 17-fold that of the control) than at 48 or 72 h. This significant increase in jute *CCoAOMT* expression indicates its potential functional value in drought resistance. Hence, it is essential to thoroughly investigate the function of jute *CCoAOMTs* in lignin deposition under drought stress, particularly in stem tissues.

We observed that salt stress (24 h) induced *CCoAOMT* expression in both jute species but at different levels, suggesting that increased monolignol biosynthesis is one of the plant’s strategies to avoid salt damage. The expression of *CCoAOMTs* showed a substantial increase in *C. olitorius*, followed by a noticeable decline along the saline stress treatment (48 h) in *C. capsularis*. Additionally, the transcript levels of *CCoAOMTs* were initially, slightly reduced in *C. olitorius* and subsequently returned to the control levels at 72 h after salt stress. In this study, five of 12 *Co.CCoAOMTs* were found to be up-regulated or down-regulated under both drought and salt stresses, while the remaining genes showed similar transcript levels as those of control plants. Similarly, three of the eight *Cc.CCoAOMTs* were up-regulated and down-regulated under the same stresses. Furthermore, some genes (*CCoAOMT1*/*2*) up-regulated in drought-stressed stems were also induced in salt-stressed stems (24 h). In contrast, the majority (78% of *Cc.CCoAOMTs* and 83% of *Co.CCoAOMTs*) of the *CCoAOMTs* of the two jute species exhibited different transcript levels under various stress conditions, suggesting that these genes may be involved in the communication between distinct signal transduction pathways. Taken together, these results suggest that jute *CCoAOMTs* may participate in the stress response to drought and salt treatment. These results also provide a molecular basis for functional characterization of *CCoAOMT* genes by overexpression or silencing. Furthermore, additional proteomic assays of CCoAOMTs under abiotic stress may also help to reveal their physiological functions.

## Conclusion

We identified 66 *CCoAOMT* genes from 12 plant genomes including 20 *CCoAOMTs* in *Corchorus olitorius* and C. *capsularis*. The study indicated that *CCoAOMTs* underwent gene restructuring under dissimilar evolutionary dynamics in jute and angiosperms. Phylogenetic analysis revealed that gene structures and amino acid sequences of CCoAOMTs are conserved between *C. capsularis* and *C. olitorius*, and *CCoAOMT* genes of *Corchorus* have the closest evolutionary relationship with *Theobroma cacao* and *Gossypium raimondii*. Furthermore, the expression level of *CCoAOMT2* indicated that this gene plays a leading role in lignin biosynthesis of jute. Meanwhile, we found out that *CCoAOMTs* had positive correlation with lignin content (CL variety), and *CCoAOMTs* were also involved in the regulation of salt and drought resistance on jute. These findings revealed evolutionary, expression and function aspects of the *CCoAOMT* genes in jute and identify genes that may be useful for genetic manipulation on improving fiber quality of jute.

## Methods

### Plant materials and culture conditions

Six varieties were used in this study, namely three for C. *capsularis* (J179, LB, and QY) and three for *C. olitorius* (F5, KY, and CL). These were provided by the Key Laboratory of Ministry of Education for Genetics, Breeding and Multiple Utilization of Crops (Fuzhou, China), and Institute of Bast Fiber Crops, Chinese Academy of Agriculture Sciences (Changsha, China). The seeds were sterilized with 5% NaOCl for 10 min and rinsed three times with sterilized water before planting in pots containing nutrient soil, and the diameter and height of the pots are both 20 cm. The plants were grown for up to 90 days in a greenhouse with temperatures of 30/26 °C (day/night) in a 16/8 h light/dark cycle. Samples were taken from the stems of jute plants at five stages (15, 30, 45, 60, and 90 "DAS"). Additionally, the whole jute stem of 60 DAS was separated into three parts (top, middle, bottom) according to the plant height, and the middle stem of each part was taken as the samples to identify the expression levels of *CCoAOMT* genes. For abiotic stress treatment, two jute cultivars, the drought-sensitive *C. olitorius* F5 and the drought-tolerant *C. capsularis* J179 were used. The seedlings were cultivated in 1/4 Hoagland solution in the same greenhouse. The seedlings were subjected to abiotic stresses at 30 DAS. The plants were exposed to 10% (w/v) PEG6000 and 200 mM sodium chloride, and collected at three time points (24, 48, and 72 h) [64]. Subsequently, the samples were quickly placed in liquid nitrogen and stored at -80 °C for RNA extraction.

### RNA isolation and first strand cDNA synthesis

Total RNA was isolated from nearly 100 mg of fresh stem tissue using the OMEGA isolation kit (R6827-01, USA) and the Huayueyang Biotechnology isolation kit (Beijing, China), according to the manufacturers’ instructions. Genomic DNA contamination was removed by RNase-free DNase I (TaKaRa, Japan) treatment. The quality of RNA was assessed using a NanoDrop 2000 spectrophotometer (NanoDrop, Thermo Fisher Scientific). The integrity of RNA was evaluated by 2.0% agarose gel electrophoresis. Lastly, RNA specimens with an A260/A230 ratio of more than 1.8 and an A260/A280 ratio between 1.8 and 2.2 were used for further analysis. Subsequently, using the PrimeScript RT reagent kit (TaKaRa, Japan), first-strand cDNA was synthetized from 1 μg of complete RNA in a 20μL reaction mixture, according to the manufacturer’s guidelines. The final cDNA was diluted in water for "qRT-PCR".

### Identification of the *CCoAOMT* gene family in jute

The genome sequence of jute was downloaded from the NCBI database [1]. To identify the *CCoAOMT* gene family in jute, the sequences of *A. thaliana* and rice CCoAOMT proteins [61, 65] were used as queries to search against the *C. capsularis* and *C. olitorius* genomes using tBLASTn and BLASTp on the NCBI database [66] with default parameters. All hits were confirmed using the InterProScan program [67] to verify the existence of the protein domain.

### Cloning of jute *CCoAOMT* genes

To clone the full gene and open reading frame (ORF) sequences of the jute *CCoAOMT* genes, pairs of primers (Additional file 19) were designed according to the coding sequences (CDS) of *CCoAOMTs* reported in GenBank resources for jute. Forward and reverse primers were designed to align to the opposite endings of the ORF. DNA was extracted from leaf samples of young jute plants using the TIANcombi DNA Lyse & Det PCR Kit (Tiangen, China), according to the manufacturer’s guidelines. PCR products derived from genomic DNA amplification were cloned into the pMD19-T cloning vector (TaKaRa Biotechnology, Dalian, China) and subsequently sequenced by BGI Tech Solutions Co., Ltd. (BGI-Tech). The obtained sequences were BLAST-searched against the NCBI database for validation.

### BLAST searches of *CCoAOMT* gene family members in eight representative dicot and monocot plant species

The sequences of seven known *CCoAOMT* genes from *A. thaliana*) [59] were utilized as queries to identify the complete sequences of *CCoAOMT* genes in rice (*Oryza sativa*), ramie (*Boehmeria nivea*), soybean (*Glycine max*), cotton (*Gossypium raimondii*), kenaf (*Hibiscus cannabinus*), flax (*Linum usitatissimum*), cacao (*Theobroma cacao*), and Amborella (*Amborella trichopoda)*. Candidate sequences that fulfilled the criteria of a probability score < 10^−4^ and a similarity score > 50.0 were selected. These candidate CCoAOMT sequences were further confirmed by searching against the annotated databases of Phytozome [68] and NCBI using BLASTX and BLAST. Moreover, candidate CCoAOMT proteins were assessed by investigating their conserved domains using the CD-Search and InterPro tools [69, 70]. All Cc.CCoAOMT and Co.CCoAOMT proteins share a conserved domain which is “AdoMet_MTases” with the exception of Cc.CCoAOMT2 and Co.CCoAOMT2 proteins which share “PLN02589” as their conserved domain. Using the ExPASy Translate tool [71], the confirmed DNA sequences were translated into proteins. Additionally, the theoretical isoelectric point (pI) and molecular weight (Mw) of the proteins were calculated using the ExPASy Compute pI/Mw tool [72]. Prediction of subcellular localization was conducted using the Prediction Servers of Signal P5.0 Server [73], PlantmPLoc [74], MitoProt [75], and ChloroP [76]. The CCoAOMT protein motifs were assayed using the online tool MEME Suite [77] with the frameworks of minimum motif width being 6, maximum motif number being 15, maximum motif width being 50 and dissemination of motif occurrences being one per sequence or Zero. The gene schematic structures were drawn, using the Gene Structure Display Server [78, 79].

### Phylogenetic analysis

The evolutionary history of CCoAOMTs in 12 plant species was deduced using the neighbor-joining method [80]. The ratio of replicate trees was displayed next to the branches [81]. Evolutionary distances were calculated using the Poisson correction method [82] and were expressed as the number of amino acid substitutions per site. Sixty-six amino acid sequences were used in the analysis [83]. By applying the Neighbor-Joining and BioNJ algorithms to a matrix of pairwise distances, the initial trees for the heuristic search were obtained automatically. Subsequently, the topology showing a superior log-likelihood value was chosen. Using the Easy_KaKs calculation program [84], the non-synonymous (Ka) and synonymous (Ks) substitution ratios of eight pairs of orthologous *CCoAOMT* genes were computed. Finally, Fisher’s exact test was used to confirm the validity and compute the p-value of the Ka and Ks ratios [85] and the divergence time (T) was computed by using this formula: $\mathrm{T}=\mathrm{Ks}/(2\times 6.1\times {10}^{^-9})\times {10}^{^-6}\mathrm{Mya}$ [86]. Utilizing MCScanX analysis [87], collinearity relationships of *CCoAOMT* genes and classifier program were used to sort gene duplication types. The identified collinear gene pairs were mapped to their respective locus in the jute genome in a circular diagram using Circos 0.69 [88].

### Analysis of gene expression by qRT-PCR

One microgram of total RNA from each of the samples collected from plants at various developmental stages subjected to different treatments was reverse-transcribed to cDNA using the Reverse Transcriptase Kit (TaKaRa) in a 20uL reaction mixture, according to the manufacturer’s guidelines. The resulting cDNA was diluted for qRT-PCR analysis. Based on the annotated sequences of *CCoAOMTs*, qPCR primers (Additional file 20) were designed using the PrimerQuest tool at Integrated DNA Technologies [89]. The expression patterns of 12 *Co.CCoAOMT* genes and eight orthologous *Cc.CCoAOMT* genes were compared across the different developmental stages. qRT-PCR was performed using the Multicolor Real-Time PCR Detection System (Bio-Rad). The qRT-PCR reactions were incubated at 94 °C for 2 min, and then subjected to 39 cycles of 94 °C for 5 s, 65 °C for 5 s, and 72 °C for 5 s. Melting curve analysis was conducted to verify qPCR specificity. For the developmental stage group, ubiquitin extension protein (*UBI*) was selected as a reference gene. For the salt stress group, actin 7 (*ACT7*) was selected as an internal control; finally, for drought stress, ubiquitin-conjugating enzyme-like protein (*UBC*) was used as a reference gene (Additional file 20) [90]. The 2^−ΔΔCT^^−^method [91] was utilized to calculate relative expression levels for each *CCoAOMT* gene.

### Lignin content and composition of developing jute fibers

The measurement of lignin content was conducted on jute stem samples (J179, QY, LB, F5, KY, and CL) collected at 15, 30, 45, 60, and 90 DAS. Three biological replicates were used for each sample. The determination of lignin constituents in jute threads was carried out through the acetyl bromide method [92, 93]. The results were expressed as mg lignin g^−1^ cell wall. SPSS20.0 was used to perform statistical analysis of lignin content data for 6 genotypes and 5 developmental stages, as well as the correlation between lignin content and *CCoAMOT2* expression level.

## Supplementary Information

**Additional file 1.** The result of PCR amplification of cloned *CCoAOMT *genes in jute.

**Additional file 2.** Basic information of *CCoAOMT* genes in 12 plant species.

**Additional file 3.** (A) The similarity between CCoAOMT proteins in *Corchorus olitorius* was calculated via NCBI BLASTP. (B) The similarity between CCoAOMT proteins in *Corchorus capsularis *was calculated via NCBI BLASTP.

**Additional file 4.** Illustrative representation of the conserved motifs in CCoAOMT proteins.

**Additional file 5.** Divergence between two pairs of tandem duplicated genes (*Co.CCoAOMT*3a & *Co.CCoAOMT*3b, *Co.CCoAOMT*5a & *Co.CCoAOMT*5b).

**Additional file 6.** The Ka/Ks analysis for *CCoAOMT* gene pairs in jute.

**Additional file 7.** Amino acid sequences of CCoAOMT in 11 species.

**Additional file 8.** A schematic diagram for the relationship of the 5 groups of the phylogenetic tree constructed by NJ method.

**Additional file 9.** Divergence time among the five groups of *CCoAOMT* gene family in jute.

**Additional file 10.** The non-synonymous (Ka) and synonymous (Ks) substitution ratio of *CCoAOMTs *from 2 representive plants.

**Additional file 11.** The exon number of *CCoAOMTs* in jute and other ten representative species.

**Additional file 12.** The proportion of the same number of exons in all *CCoAOMTs*.

**Additional file 13.** Overall collinearity relationships of *CCoAOMT* genes on the jute genome.

**Additional file 14.** Expression profile of *CCoAOMT2* in six cultivars of jute at each developmental stage.

**Additional file 15.** Change in expression level of *CCoAOMT* genes that were highly expressed in different stages of stem development in jute.

**Additional file 16.** The expression levels of *CCoAOMT *genes under different parts (top, middle, bottom) of 60 DAS of the stem of C. *capsularis* and C. *olitorius*.

**Additional file 17.** The sum of normalized expression of all *CCoAOMT *genes from each developmental stage.

**Additional file 18.** Correlation coefficient between lignin content and *CCoAOMT2* expression level under different varieties (F5, CL, KY, J179, QY, and LB) and different developmental stages (15 DAS, 30 DAS, 45 DAS, 60 DAS and 90 DAS).

**Additional file 19.** PCR primer sequences of *CCoAOMT* genes in jute.

**Additional file 20.** Jute *CCoAOMT* gene-specific primers and reference genes used for qRT-PCR analysis.

## Data Availability

The CDS sequences of *CCoAOMT* for *C. capsularis* and *C*. *olitorius* are accessible through the NCBI under the accession numbers of BankIt2603300 and BankIt2603415. The data of CCoAOMT Protein sequences from *C. capsularis* and *C*. *olitorius* are in UniProtKB with accession numbers of A0A1R3GF10 and A0A1R3K4I7. The other data supporting the conclusions of this article are within the paper.

## Abbreviations

CDS: Coding sequence
DAS: Days after sowing
LCA: Last common ancestor
WGD: Whole-genome duplication
J179: Jute-179
LB: Lubinyuanguo
QY: Qiongyueqing
F5: Funong No.5
KY: Kuanyechangguo
CL: Cvlv
PEG: Polyethylene glycol
NaCl: Sodium chloride
qRT-PCR: Quantitative reverse transcription polymerase chain reaction
