# Supplementary material for: Evolution and expression analysis of the caffeoyl-CoA 3-O-methyltransferase (CCoAOMT) gene family in jute (Corchorus L.)
Source: BMC Genomics. 2023 Apr 17;24:204. doi: 10.1186/s12864-023-09281-w (PMC10111781; doi:10.1186/s12864-023-09281-w)
Supplement: Supplementary file 1 — Additional file 1. The result of PCR amplification of cloned CCoAOMT genes in jute. [file 12864_2023_9281_MOESM1_ESM.docx]

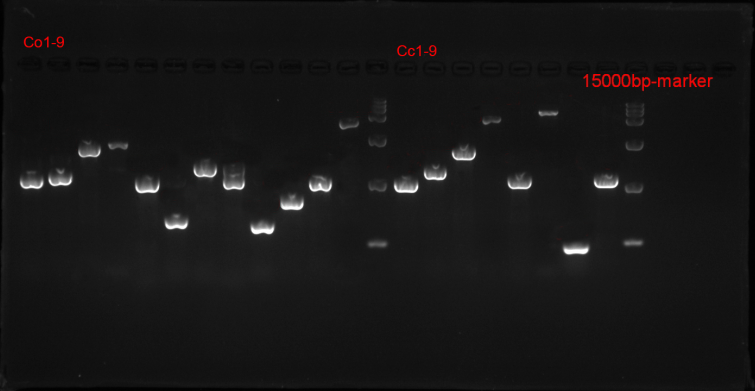


**Additional file1: The result of PCR ampliﬁcation of cloned *CCoAOMT* genes in jute. Note: Co1-9 represent *Co.CCoAOMT1-9* and Cc1-9 represent *Cc.CCoAOMT1-9*.**
