# Supplementary material for: Evolution and expression analysis of the caffeoyl-CoA 3-O-methyltransferase (CCoAOMT) gene family in jute (Corchorus L.)
Source: BMC Genomics. 2023 Apr 17;24:204. doi: 10.1186/s12864-023-09281-w (PMC10111781; doi:10.1186/s12864-023-09281-w)
Supplement: Supplementary file 4 — Additional file 4. Illustrative representation of the conserved motifs in CCoAOMT proteins. [file 12864_2023_9281_MOESM4_ESM.pdf]

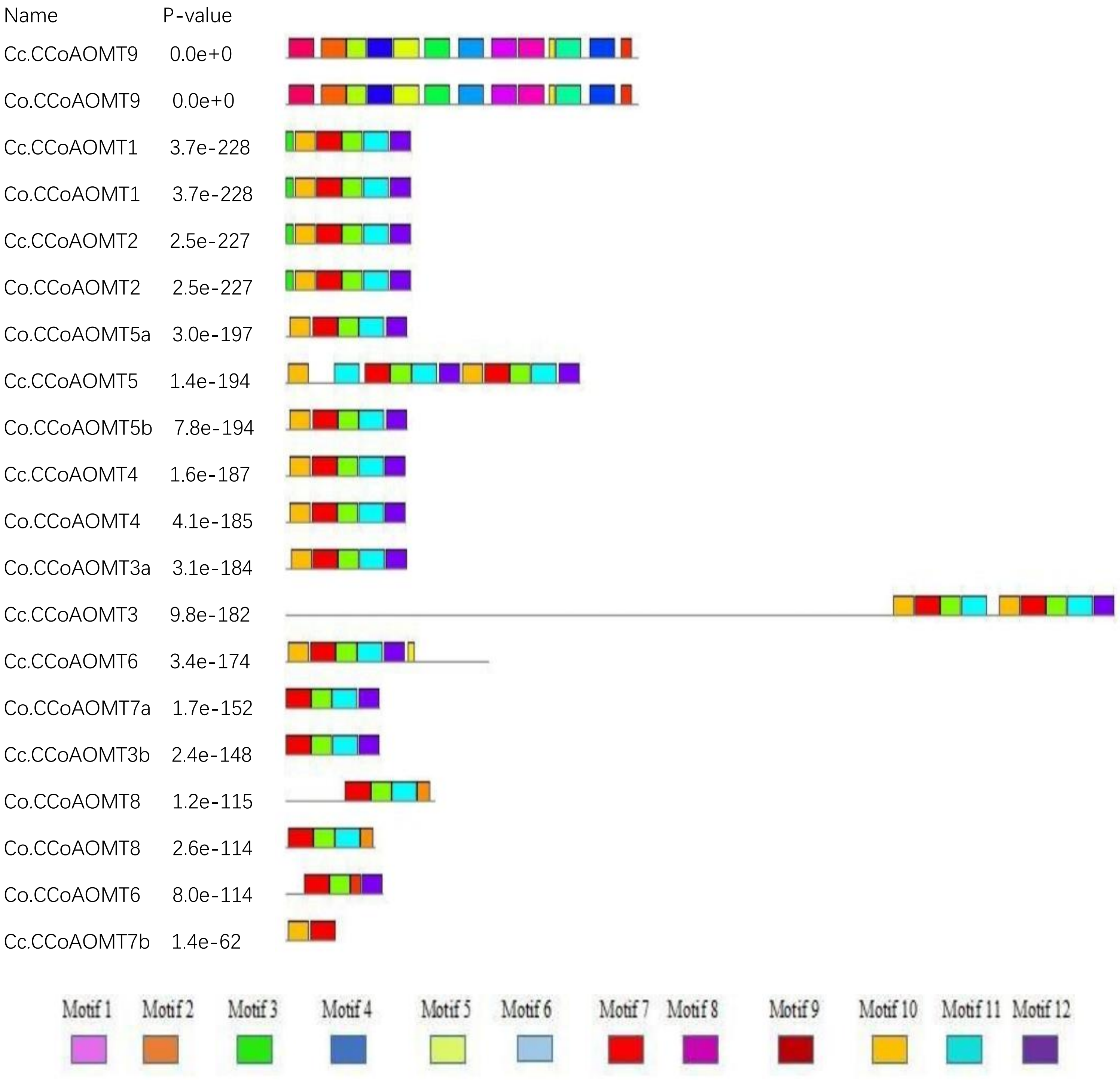

Additional file 4: Illustrative representation of the conserved motifs in CCoAOMT proteins.
Note: The 12 motifs are written at the bottom of the figure.
