# Supplementary material for: Evolution and expression analysis of the caffeoyl-CoA 3-O-methyltransferase (CCoAOMT) gene family in jute (Corchorus L.)
Source: BMC Genomics. 2023 Apr 17;24:204. doi: 10.1186/s12864-023-09281-w (PMC10111781; doi:10.1186/s12864-023-09281-w)
Supplement: Supplementary file 5 — Additional file 5. Divergence between two pairs of tandem duplicated genes (Co.CCoAOMT3a & Co.CCoAOMT3b, Co.CCoAOMT5a & Co.CCoAOMT5b). [file 12864_2023_9281_MOESM5_ESM.docx]

**Additional file 5: Divergence between two pairs of tandem duplicated genes (*Co.CCoAOMT3a & Co.CCoAOMT3b, Co.CCoAOMT5a & Co.CCoAOMT5b*).**

| Gene1 | Gene2 | Ka | Ks | Ka/Ks | Duplication type | Divergence time | P-Value (Fisher) |
| --- | --- | --- | --- | --- | --- | --- | --- |
| *Co*.CCoAOMT3a | *Co.CCoAOMT3b* | 0.207 | 1.760 | 0.118 | Tandem | 144.23 | 4.25799E-26 |
| *Co.CCoAOMT5a* | *Co.CCoAOMT5b* | 0.025 | 0.103 | 0.246 | Tandem | 8.43 | 0.000266941 |

Note: the unit of divergence time is Mya (Million years ago).
