# Supplementary material for: Evolution and expression analysis of the caffeoyl-CoA 3-O-methyltransferase (CCoAOMT) gene family in jute (Corchorus L.)
Source: BMC Genomics. 2023 Apr 17;24:204. doi: 10.1186/s12864-023-09281-w (PMC10111781; doi:10.1186/s12864-023-09281-w)
Supplement: Supplementary file 6 — Additional file 6. The Ka/Ks analysis for CCoAOMT gene pairs in jute. [file 12864_2023_9281_MOESM6_ESM.docx]

**Additional file 6: The Ka/Ks analysis for *CCoAOMT* gene pairs in jute.**

| Gene 1 | Gene 2 | Ka | Ks | Ka/Ks | Purifying Selection | Divergence time (Mya) |
| --- | --- | --- | --- | --- | --- | --- |
| *Cc.CCoAOMT1* | *Co.CCoAOMT1* | NA | 0.068633 | NA | yes | 5.625647541 |
| *Cc.CCoAOMT2* | *Co.CCoAOMT2* | NA | 0.0685826 | NA | yes | 5.62152459 |
| *Cc.CCoAOMT3* | *Co.CCoAOMT3a* | 0.0641227 | 0.201982 | 0.317468 | yes | 16.55590164 |
| *Cc.CCoAOMT3* | *Co.CCoAOMT3b* | 0.0238847 | 0.10505 | 0.227365 | yes | 8.610655738 |
| *Cc.CCoAOMT4* | *Co.CCoAOMT4* | 0.0219557 | 0.0880939 | 0.249231 | yes | 7.220811475 |
| *Cc.CCoAOMT5* | *Co.CCoAOMT5a* | 0.0272444 | 0.132564 | 0.205519 | yes | 10.86590164 |
| *Cc.CCoAOMT5* | *Co.CCoAOMT5b* | 0.025373 | 0.0956838 | 0.265176 | yes | 7.842934426 |
| *Cc.CCoAOMT6* | *Co.CCoAOMT6* | 0.0578587 | 0.117728 | 0.491461 | yes | 9.649836066 |
| *Cc.CCoAOMT8* | *Co.CCoAOMT8* | 0.0252516 | 0.124234 | 0.203259 | yes | 10.18311475 |
| *Cc.CCoAOMT9* | *Co.CCoAOMT9* | 0.0144612 | 0.0432255 | 0.334552 | yes | 3.54307377 |
