# Supplementary material for: Evolution and expression analysis of the caffeoyl-CoA 3-O-methyltransferase (CCoAOMT) gene family in jute (Corchorus L.)
Source: BMC Genomics. 2023 Apr 17;24:204. doi: 10.1186/s12864-023-09281-w (PMC10111781; doi:10.1186/s12864-023-09281-w)
Supplement: Supplementary file 7 — Additional file 7. Amino acid sequences of CCoAOMT in 11 species. [file 12864_2023_9281_MOESM7_ESM.docx]

Additional file 7: Amino acid sequences of CCoAOMT in 11 species.

>AtCCoAOMT1

MATTTTEATKTSSTNGEDQKQSQNLRHQEVGHKSLLQSDDLYQYILETSVYPREPESMKELREVTAKHPWNIMTTSADEGQFLNMLIKLVNAKNTMEIGVYTGYSLLATALALPEDGKILAMDVNRENYELGLPIIEKAGVAHKIDFREGPALPVLDEIVADEKNHGTYDFIFVDADKDXYINYHKRLIDLVKIGGVIGYDNTLWNGSVVAPPDAPMRKYVRYYRDFVLELNKALAADPRIEICMLPVGDGITICRRIS

>AtCCoAOMT2

MAKDEAKGLLKSEELYKYILETSVYPREPEVLRELRNITHNHPQAGMATAPDAGQLMGMLLNLVNARKTIEVGVFTGYSLLLTALTLPEDGKVIAIDMNRDSYEIGLPVIKKAGVEHKIDFKESEALPALDELLNNKVNEGGFXXAFVDADKLNYWNYHERLIRLIKVGGIIVYDNTLWGGSVAEPDSSTPEWRIEVKKATLELNKKLSADQRVQISQAALGDGITICRRLY

>AtCCoAOMT3

MSTGLALNRCSVSVCRTAVTLLNRPTVSVARSLKFSRRLIGNCSIAPADPYVVADDDKYGNKQVISLTPRLYDYVLSNVREPKILRQLREETSKMRGSQMQVSPDQAQLLAMLVQMLAAERCIEVGVYTGYSSLAVALVLPESGCLVACERDSNSLEVAKRYYELAGVSHKVNVKQGLAAESLKSMIQNGEGASYDFAFVDADKRMYQDYFELLLQLVRVGGVIVMDNVLWHGRVSDPMVNDAKTISIRNFNKKLMDDKRVSISMVPIGDGMTICRKR

>AtCCoAOMT4

MTTFSTSFLFLLLVFCLIGSLAADDLQHKSGRDVCSGGSDLRTPDIRLNRPTDSVVGNCPTEASPLVMADDEKYGNKMVISLTPRLYDYVLNNVREHEILKQLREETAISQIQVSPDQAQLLAMLVEILGAKRCIEVGVYTGYSSLAVALVLPESGRLVACDKDANALEVAKRYYELAGVSHKVTVKHGLAAESLMSMIQNGEESSYDFAFLDADKAMYQEYFESLLRLVRVGGVIVIDNVLWHGWVADSTVNDERTISLRNFNKKLMDDQRVSISMVSIGDGMTICRKR

>AtCCoAOMT5

MDGRLPDKGILKSEALKQYIMETTAYPREHELLKELREATIQRYGNLSEMGVPVDESLFLSMLVKIINAKNTIEIGVFTGYSLFTVALALPEDGRITAIDIDQAGYNLGLEFMKKAGVDHKINFIQSDAVRGLDQLLNGKQEYDFAFVDADKTNYVYFLEKLLKLVKVGGIIAFDNTLWFGTLIQKENEVPGHMRAYREALLEFNKILARDPRVEIAQISIGDGLTLCRRLI

>AtCCoAOMT6

MANEIPTKGILKSEALKQYIMETSAYPREHELLKELRKATVQKYGNLSEMEVPVDEGHFLSMLVKIMNAKNTIEIGVFTGYSLLTTALALPEDGRITAIDIDKEAYEVGLEFIKKAGVDHKINFIHSDGLKALDQLVNDKCEFDFAFADADKSSYVNFHERLLKLVKVGGIIAFDNTLWFGFVAEDEDGVPEHMREYRAALIEFNKKLALDPRVEVSQISIGDGITLCRRLV

>AtCCoAOMT7

MEKLLPPSKLLPPKGILKSDALKKYIFETTAYPREHEQLKKLREATVLKYGNLSEMEVPVDEGHFLSMLLKIMNAKKTIELGVFTGYSLLTTALALPHDGHVTGIDIDKEAYEMGLEFIKNAGVHHKINFIHSDCLQALDNMLSENPKPEFDFAFVDADKPNYANMHERLMKLVKVGGVIAFDNTLWSGFVAEKEENVPVHMRVNRKAFLDLNKRLAADPHVEVSQVSIGDGVTLCRRLV

>BnCCoAOMT1

TSVYPREPESMKELREITAKHPWNLMTTSADEGQFLSMLLKLINAKNTMEIGVFTGYSLLATALALPEDGKILAMDINRENYELGLPVIEKAGLAHKIDFREGPALPALDQMLEDGKYHGSFDFIFVDADKDNYLNYHKRLIELVKVGGVIGYDNTLWNGSV

>BnCCoAOMT2

TSVYPREPESMKELREITAKHPWNLMTTSADEGQFLSMLLKLINAKNTMEIGVFTGYSLLANGLALPEDGKILAMDINRENYELGLPVIEKAGLAHKIDFREGPALPALDQMIEDGKYHGSFDFIFVDADKDNYLNYHKRLIELVKVGGVIGYDNTLWNGSV

>BnCCoAOMT3

MANNGEGKQNEVSRHQEVGHKSLLQSDALYQYILETSVYPREPESMKELREITAKHPWNLMTTSADEGQFLSMLLKLINAKNTMEIGVFTGYSLLATALALPEDGKILAMDINRENYELGLPVIEKAGLAHKIDFREGPALPALDQMIEDGKYHGSFDFIFVDADKDNYLNYHKRLIELVKVGGVIGYDNTLWNGSVVAPPDAPLRKYVRYYRDFVLELNKALAADPRIEICMLPVGDGIPICRRVS

>BnCCoAOMT4

TSVYPREPESMKELREITAKHPWNLMTTSADEGQFLSMLLKLINAKNTMEIGVFTGYSLLATALALPEDGKILAMDINRENYELGLPVIEKAGLAHKIDFREGPALPALDQMIEDGKYHGSFDFIFVDADKDNYLNYHKRLIELVKVGGVIGYDNTLWNGSV

>Cc.CCoAOMT1

MAPTQAEQQTQASRHQEVGHKSLLQSDKLYQYILETSVYPREPEAMKELRELTAKHPWNLMTTSADEGQFLNMLLKLINAKNTMEIGVYTGYSLLATALALPEDGKILAMDINRENYELGLPVIQKAGVAHKIDFKEGPALPVLDQMIEAGTYHGTFDFIFVDADKDNYINYHKRLIELVKVGGVIGYDNTLWNGSVVAPPDAPLRKYVLYYRDFVLELNKALAADPRIEICQLPVGDGI

TLCRRIK

>Cc.CCoAOMT2

MATNTQEQQTQAGRHQEVGHKSLLQSDALYQYILETSVYPREPEPMKELRELTAKHPWNLMTTSADEGQFLNMLLKLINAKNTMEIGVYTGYSLLATALALPDDGKILAMDINRENYELGLPVIQKAGVAHKIEFKEGPALPVLDKLVEDEKNHGSYDFIFVDADKDNYINYHKRLIDLVKVGGLIGYDNTLWNGSVVAPPDAPLRKYVRYYRDFVLELNKALAADPRIEICMLPVGDGI

TLCRRIK

>Cc.CCoAOMT3

MLHRSFKPAKCKTALKLAIPRLKLMKNKREAQVKQLRRELAQLLESGQDQTARIRVEHVVREEKTVAAYNLLEIYCELIVARMPIIESQKNCPIDLKEAIASVVFASARCGEIPELKDVSKHFTAKYGKEFTSAALELRPNCGVGRMLVEKLSASAPDGPTKLKILTAIAEEHNIKWDPESFGAKEAKVYEDLLNLPNTVKEATKIADPPKAQASTSHYEQRTPINQVPTHDKGPPNVQAPKHMEKNDAPASVYGHSSGSPPYAKNFGNSNSSASNKMSSGTYPPNSKPYGTEHQEMEFRNSYSGNESAFSSPRQHWNMEFKDATAAAQAAAESAELASMAARAAAELSSRENITRQNSTESRMSSAHGMRNDEPHQYTASASQNEHLARRPVAQGRNSQNYGDTDRKELHNRAGQAENMYSNIVMSADKSTHGSFKSTAASSIERPSVNNQIGDAYSQRNSSEGRQVEQFSEVTTKRSSGKNGMQFLSEVHGSKNVDNHEVRVREQSSYSSSHSQLNTSTDDHDVVSNLKWQSSDYDERNSSKTRMQFVNELHDIKNSEIADYQEATIRKQSSYSSSHSSSSAFADDHDVVSNLNRQNSGNNSGEESFPFNDKGSHHRSTKETTDSYDNPSAVFDNYGSDNGGCNFDLEEEHKVHEYSMDFLSPGRKSPTHPFTSTNSWRIGQTVDSPEKSISQSHIFSEKQSTPVFDESSTSSAVASQRDDLPAAFDDYGPSSESEEEVEKSKFDRSGDTSIGSDKQNNDFHQSKTSISTPQLAEGIEGTEPFKDFSMEESKELNLRNLTGGIRNKNKLPPYSRVPQSSTIHSEEATNFTSTRTKQSSTPTAVEASVSSGSYNQEPYSRKGSVEVNRKLSTRASRQIQQDSDSSDDDSEEEEIQPYTSTEDQHDKMPSFEENKVSNLRAPIPYFGSGNSDSDQELPKTSPNSRLNTGLSRRTKASPSNSRRSSNLKTTVSSEPKVFSDYGGEKYPSLRSSNANEALPRTRPQKKDSDYWESNQQSRLAAQATTKLVSETKKSSFDGPPDYGREKYSSLRNSNADEASPRTQPQKKDSDHWESNQQSRLAARTTTKLVSETKKSSFDGPPDYGREKYSSLRNSNADEAQPRSQPQKQDSDHWESNQQSRLAARSTNKLVSETKKSSFDGPPVSSQMEQQAPTSVPKVIASEESMENTSDKQLDKPTKGLLQSTDLYEYILETSVYPREAELLKELRAATANHPECIISTAPDAGQLMGMLLKLVNAKKTIEIGVYTGYSLLLTALSIPSDGQIIAIDINSETYEIGLPIIRKAGVENKINFIESQALPVLDKLLKDKENEGSFDFAFVDADKNNYWNYHERLLKLVKVGGVIIYDNTLWIGTVARPEEEVSEDKREWRRLRAMDNEKKESTSFSKGLLQNEELYRYILETSVYPHESEYLKELRDITATHPWSIMATAPDAGQLIAMLLNLINAKKTIEVGVFTGYSLLLTALTIPEDGKILAIDLNREAYEIGLPVIRRAGVENKIDFRESAALPVLDQLLEDPGNENAFDFAFIDADKINYWNYHERLMKLVKVGGIVAYDNTLWGGTVAIPEECTPEGVREGRQRTLDFNKLLAADSHVQILLAPLGDGITICRRLH

>Cc.CCoAOMT4

MANVDNISPKCILKSDVLLEYILNTSAYPREHEQLKGLRETTAEKYPNESLMNVPVDEGQFLSMLLKLMNAKKTLEIGVFTGYSLLATALALPNDGQITAIDPQREFYELGLPFIKKAGMEHKINFIESEALKVLTEMSNNEKDKPEFDFIFVDADKPNYMKYHEYLKKLVKVGGVIAYDNTLWFGFLVQEEAQVPEPARPSRKALLELNMSLASDPSMEVCQISIGDGVTLCRRIC

>Cc.CCoAOMT5

MTDWSKKTILRNDALVQYILQTSAFPKEHEQLKELREASAQKYKELSVMNVPADEAQFLSMLLKLINAKKTLEVGVFTGYSLLATALALPQDGKGEEGSFDFIFVDAYKSDYLKFHELTLKLVKIGGIIAYDNTLWYGSVAESEKEVTEDLIKRSVMNVPADEGQFLSMLLKMMNAKKTIEIGVFTGYSLLTTALALPEDGKIIAIDPDKEAYEVGLPFIKKAGIEHKINFIPSDAFLVLNDLINSGEEGTFDFIFVDASKNDYLKFHELTVKLVKIGGIIAYDNTLFMGSVGKSEEEIKEEPMRQLRNSVMEFNSFISADPLPRVESSLLSIGDGLTLCRQAVSSLNHKSLIVQPSVLEYILEKNAYPKEHEQLKKLREVTAEKYEKKSIMNVPADEAQLLSMLLKVMNAKKTMEIGVFTGYSLLATALALPQDAQITAIDLDKEAYETGLPFIKEAGVDHKINFINSDAFLVLDDLINGGDDEGKFDFIFVDAQKKDYKKFHEQVLKLVKVGGIIAYDNTLWFGSVGYEEEKDWMPEFVWKSREFVLQFNSFLATDPRIESSLLSIGDGLTLCKRLY

>Cc.CCoAOMT6

MSAYDYRRMILKSDALQEYIYETSAYPKEHLQLKELREATIEKYQVWSAMSLPVDEAQFLSLLVKIMNAKKTMEIGVFTGYSLLTTALALPEDGQILAIDPDKEAYEFGLPYLKKAGVEHKINFVPSDAISYLNGLVNSSEEGSFDFIFVDAFKDQCLEFHEIALKFVKIGGTIGYDNTLWYDSVAQPEEEVTDEHIRSYRNFVVEFNDFVAADPRVESSIISIGDGSKTLTILDFIRTTAVCRPWKVCLKDHKPKFPVYLMLAEKEDEDTNNGMRIEEYEDIDEGDGDGVVETKTWIITEGFEIFKLDTQCRIWEKILSLGDRSLFPGNCCTFSVLAADYPNCNSNYVMNDDSHSWYRKGPGDYDIGIYNCDNKEVLQLPVSDDKQRFRLNSPHLFGSI

>Cc.CCoAOMT8

MHSAPPLSLPRMATSFTISRCSASACQRAVVFLSRTQRCHSFSPRTAKFRFLKLNHLTRNCSSSPKAPFIVADDEKYGNKQVISITPRLYDYILGNVREPQILRQLREETANMRGSQMQVSPDQAQLLAMLVQILGAERCIELGVYTGYSSLAIALALPESGCLVACERDARSLEVAKKYYALAGVSDKIIVKHGLAADALKSMILNGEACSYDFAFVDAEKKMNQEYFELLLQLVRVGGVIVIDNVLWHGKVADPLVNDAKTVSIRNFNRSLMEDNRVSISMMLKMLASETCL

>Cc.CCoAOMT9

MSSDSSQRVFQLKLDPLTGNSEWVVIEDNDELQETSNEPLLATTSYLDMLNDSYRNKAFRLAIEKTVTKPCHVLDIGAGTGLLSMMAARAMGLNGKVTACEAYLPMAKLMRKVLHRNGMTKNINLINKRSDELEVGIDIPSRADVLVSEILDSELLGEGLIPTLQHAHDKLLVENPLTVPYRAVTYGQLVESSYLWKLHDLSGNEAEGSDSIHLVPTGLDTIIDVKSRQYPMHCDAIRKE

IKLLSEPFKIFEFDFWKRPDSHGETEVQIKAIGDGNIHAVVSWWILQLDREGTVFYSTAPRWINSTANVGDRNWCDHWKQCVWLLPGKGMPVSKEEEVLLRATHTETSVSYNLNVQVPQTDRRQHDHRIGDLQLLLSPERIAAYGDSEWQLSTLAAVRNALQGKVNPLCVVADDSIFLTLLAANLSKTSHVIALFPGLRGKGAQYVRTVSKANGFSTDHVEVPEQRKACLTMYDTHGKKIDLLIGEPYYYGNEGMLPWQNLRFWKERTLLDPVLSENALVMPCKGILKACAMSLPDLWRSRRCLGELEGFEHSIVNTTLGACGELPALKEGPYLPFSIWQCGEIKELSETFTILEFDCSKPISSCYGKAQVQFNEHGICHGFVLWMDWVMDPENSIVASTGPDQRYWKQGVKLLAKPIAVGIHGSQSTSEFGSAVMEAFFDPSNSELIIKHIFS

>Co.CCoAOMT1

MAPTQAEQQTQASRHQEVGHKSLLQSDKLYQYILETSVYPREPEAMKELRELTAKHPWNLMTTSADEGQFLNMLLKLINAKNTMEIGVYTGYSLLATALALPEDGKILAMDINRENYELGLPVIQKAGVAHKIDFKEGPALPVLDQMIEAGTYHGTFDFIFVDADKDNYINYHKRLIELVKVGGVIGYDNTLWNGSVVAPPDAPLRKYVLYYRDFVLELNKALAADPRIEICQLPVGDGI

TLCRRIK

>Co.CCoAOMT2

MATNTQEQQTQAGRHQEVGHKSLLQSDALYQYILETSVYPREPEPMKELRELTAKHPWNLMTTSADEGQFLNMLLKLINAKNTMEIGVYTGYSLLATALALPDDGKILAMDINRENYELGLPVIQKAGVAHKIEFKEGPALPVLDKLVEDEKNHGSYDFIFVDADKDNYINYHKRLIDLVKVGGLIGYDNTLWNGSVVAPPDAPLRKYVRYYRDFVLELNKALAADPRIEICMLPVGDGI

TLCRRIK

>Co.CCoAOMT3a

MENTSDQQLDKPTKGLLQSTDLYEYILETSVYPREPELLKELRDATANHPGCIMSTAPDAGQLMGMLLKLVNAKKTIEIGVYTGYSLLLTALSIPSDGQIIAIDINRETYEIGLPIIRKAGVENKINFIESQALPVLDKLLQDKENEGSFDFAFVDADKNNYLNYHERLLKLVKVGGVIIYDNTLWIGTVARPEEAVSEDKREWRRSVMEFNKSISADGRLEISLAPLGDGMTICRRIC

>Co.CCoAOMT3b

MATAPDAGQLIAMLLNLINAKRTIEVGVFTGYSLLLTALTIPEDGKILAIDLNREAYEIGLPVIRKAGVENKIDFIESAALPVLDQLLEDPGNENAFDFAFIDADKINYWNYHERLMKLVKVGGIVAYDNTLWGGTVAMPEECSPEIMREGRQRTLDFNKLIAADSHVQILLAPLGDGITICRRVH

>Co.CCoAOMT4

MANVDNIPPKCILKSDALLEYILNTSAYPREHEQLKGLREATAEKYPNESLMNVPVDEGQFLSMLLKLMNAKKTLEIGVFTGYSLLATALALPDDGQITAIDPKREFYELGLPFIKNAGMEHKINFIESEALKVLTEMSSNEKDKPEFDFIFVDADKPNYMKYHEYLKKLVKVGGVIAYDNTLWFGFLVQEEAQVPEPARAPRKALLELNMNLASDLSMEVCQISIGDGVTLCRRIS

>Co.CCoAOMT5a

MAEAVSSLNHKSLIVQPPVLEYILEKNAYPKEHEQLKKLRKVTAEKYEKDKSIMNVPADEAQFLSMLLKVMNAKKTMEIGVFTGYSLLATALALPQDGQITAIDLDKEAYETGLPFIKEAGVEHKINFINSDAFLVLDDLINGGDEGNFDFIFVDAQKKDYMKFHEQVLKLVKVGGIIAYDNTLWFGSVGYEEEKDWMPELIWESREFVVQFNSFLVTDPRIESSLLSIGDGLTLCRRLY

>Co.CCoAOMT5b MAEAVSTLNHKSLIVQPPVLEYILEKNAYPKEHEQLKKLREVTAEKYEKNKSIMNVPADEAQFLSMLLKVMNAKKTMEIGVFTGYSLLATALALPQDGQITAIDMDKEAYETGLPFIKEAGVDHKINFINSDAFLVLDDLINGGDEGNFDFIFVDAQKKDYKKFHEKVLKLVKVGEIIAYDNTLWFGSVGYEEEKDWMPEFVWKSREFIVEFNSFLATDPRIESSLLSIGDGLTLCRRLY

>Co.CCoAOMT6

MLSKSIFYETSAYPKEHQQLKELREATIEKYQVWSAMSLPVDEAQFLSLLVKIMNAKKTMEIGVFTGYSLLTTALALPEDARILAIDPDKEAYEFGLPYLKKAGVEHKINFVPSDAISYLNGLVNSVGGTIGYDNTLWYGSVAQAEEEVTDEHIRSYRNFVFEFNDFVAADPRVESSIISIGDGITLCRRLY

>Co.CCoAOMT7a

MNVPADEAQFLSMLLKLINAKKTLEVGVFTGYSLLATALALPQDGKVIAIDPDNEAYETGAPFIKKAGMQHKVKFFPSDAFLVLDDLLKNGEEGSFDFIFVDAYKSDYLKFHELTLKLVKVGGIIAYDNTLWYGSVAESEKEVTEDLIKRYRNFVVEFNSFIAADPRVDSSLLSIGDGLTLCRRLY

>Co.CCoAOMT7b MADWFKKTILSNDALQEYILETSAYPKEHQQLKEIRKATVDKYKEWSVMNVPADEGQFLSMLLKMINAKKTIEIGVFTGYSLLTTALALPEDGKVNFYI

>Co.CCoAOMT8

MRGSQMQVSPDQAQLLAMLVQILGAERCIELGVYTGYSSLAIALALPESGCLVACERDARSLEVAKKYYALAGVSDKVIVKHGLAADALKSMILNGEVCSYDFAFVDAEKRMTQEYFELLLQLVRVGGVIVIDNVLWHGKVADPLVNDAKTVSIRNFNRTLMEDKRVSISMDICDWE

>Co.CCoAOMT9

MSSDSSQRVFQLKLDPLSGNSEWVVIEDNDELQETSNESLLATTSYLDMLNDSYRNKAFRLAIEKTVTKPCHVLDIGAGTGFLSMMAARAMGLNGKVTACEAYLPMAKLMRKVLHRNGMTKNINLINKRSDELEVGIDIPSRADVLVSEILDSELLGEGLIPTLQHAHDKLLVENPLTVPYRAVTYGQLVESSYLWKLHDLFGNEAEGSDGIHLVPTGLDTIIDVKSRQYPMHCDAIRKE

IKLLSEPFKIFEFDFWKRPDSQGETEVQIKAIGDGNIHAVVSWWILQLDREGTVFYSTAPRWINSTANVGDRNWCDHWKQCVWLLPGKGMPVSKEEEVLLQATHTETSVSYNLIVQVPQTDRRQHDHRIGDLQLLLSPERIAAYGDSEWRLSMLAAVRNALQGKVNPLCVVADDSIFLTLLAANLSKTSHLKALFPGLRGKGAQYVRTVSKANGFSMDHVEVPEQRKACLTMHDTHEKKIDLLIGEPYYYGNEGMLPWQNLRFWKERTLLDPVLSEDALVMPCKGILKACAMSLPDLWSSRRCLGELEGFDHSIVNTTLGACGELPALKEGPYLPFSIWQCGEIKDLSETFTILEFDCSKPISSCYGKAQVQFTEHGICHGFVLWMDWVMDAENSIIASTGPDQRYWKQGVKLLAKPIAVGIHGSQSTSEFGSAVMEAFFDPSNSELIIKHTFSMASNSRVVLDALLLEYKSEFAGFSSLVDVAGGTGTAISKIVEANPHISGINFDLPHVVATAPKYPGVVNVGGDMFSSIPSADGVFMKWILHDWKDEDCVKILKQCRKALPSKSGKLIIVDVVLHLKEDTSAFADS

>GmCCoAOMT1

MTLIKELEQQPNQIAGHKELAHKSLLQSDALYQYILETSVYPREHESLKELRELTEKHPWNLMATPPDEGQLLGMLLKLINAKNTMEIGVFTGYSLLSTALALPSDGKAGVAHKIDFREGPALPLLDQLIKDEKNKGAFDFIYVDADKDNYLNYHKRVIELVKVGGLVGYDNTLWNGSVVAPPDAPLMDYVKYYRDFVMELNKALALDSRVEICQLPVGDGITLCRRII

>GmCCoAOMT2

MSSNPVILQSENLTKYILETSVYPREEETLKELRNATASHPWGFMGAAPDAGQLMTLLLKLLNAKKTIEVGVFTGYSLLLTALTIPDDGKIIALDPDREAYEIGLPFIKKAGVEHKIDFIESPALPVLDKLIEDPSNKESFDFAFVDADKDNYWNYHERLLKLVKIGGLIIYDNTLWGGTVAWPEEDVPAPKRKFRQAALAFNKAIADDSCVEISAVSIGDGFTICRRAH

>GmCCoAOMT3

MDNISKPEVILQSEGLLKYILETGVYPREAEILKELRNATAEHPLGFMGAAPDAGQLMAMLLKLLNAKKTIEVGVFTGYSLLLTALTIPNDGKIIAMDPDRKAYEIGLPFIKKAGVEHKIDFIECPALPVLDKLLEEPANEGSFDFAFIDADKNNYWNYHERLIKLVKIGGLVAYDNTLWGGTVALPEKAVSEPKREWRRLSLAFNKAISKDCRVQIAFLSIGDGVIICMRVR

>GmCCoAOMT4

MAEQNQNQTTEAGRHQEVGHKSLLQSDALYQYILETSVYPREPESMKELRELTAKHPWNIMTTSADEGQFLNMLLKLINAKNTMEIGVYTGYSLLATALALPEDGKILAMDINRENYELGLPVIKKAGVDHKIEFREGPALPVLDEMIKDEKNHGSYDFIFVDADKDNYLNYHKRLIELVKVGGVIGYDNTLWNGSVVAPPDAPLRKYVRYYRDFVLELNKALAVDPRIEICMLPVGDGITICRRIK

>GmCCoAOMT5

MAEEERHCKSKRGLTKHKMSSNPVILQSVNLTKYILETSVYPREEETLKELRKATAGHPWGFMGAAPDAGQLMTLLLKLLNAKKTIEVGVFTGYSLLLTALTIPDDGKIIALDPDREAYEIGLPFIKKAGVEHKIDFIESPALPVLDKLLEDPSNKESFDFAFVDADKDNYWNYHERLLKLVKIGGLIIYDNTLWGGTVAWPEEDVPVPKRKLRQATLAFNKAIADDSRVEISVVSIGDGFTICRRAH

>GmCCoAOMT6

MPKPCCSMHKHYYKTNLANPGPDANRSNQTSHRFEPAFSFLISITLLHPPTSSSSNYQLFQKGEEKERKQNAQRIIIAMAEQNQNQTTEAGRHQEVGHKSLLQSDALYQYILETSVYPREPESMKELRELTAKHPWNIMTTSADEGQFLNMLLKLINAKNTMEIGVYTGYSLLATALALPEDGKILAMDINRENYELGLPVIKKAGVDHKIEFREGPALPVLDEMVKDEKNHGSYDFIFVDADKDNYLNYHKRLIELVKVGGVIGYDNTLWNGSVVAPPDAPLRKYVRYYRDFVLELNKALAVDPRIEICMLPVGDGITICRRIK

>GmCCoAOMT7

MENIKDPSIYRNPVILQSEDLTKYILETAVYPREPAPLKELREATNNHPWGFIATLPEAGQLMTLLLKLLNPKKTIEVGVFTGYSLLLTALNIPHDGKITAIDINRKTYEVGLPVIKKAGVEHKIDFIESPALPILDKLLEDPANEGSFDFAFIDADKENYVNYHERLIKLVKIGGLLVYDNTLWGGRVCWPEDKVPPHARSGRDAAIEFNKTITNDSRVEFALTSVGDGLNICRRVAI

>GrCCoAOMT1

MATNKTEEQQQQSQAGRHQEVGHKSLLQSDALYQYILETSVYPREPEPMKELREITAKHPWNLMTTSADE

GQFLNMLLKLINAKNTMEIGVYTGYSLLATALALPDDGKIFAMDINRENYELGLPVIQKAGVAHKIDFKEGPAMPVLDELVQDEKNHGSFDFIFVDADKDNYLNYHKRLIELVKVGGLIGYDNTLWNGSVVAPPDAPLRKYVRYYRDFVLELNKALAVDPRIEICMLPVGDGITLCRRLK

>GrCCoAOMT2

MATNTTQEQQSEAGRHQEVGHKSLLQSDALYQYMLETSVYPREPEPMKELRELTAKHPWNLMTTSADEGQFLNMLLKLINAKNTMEIGVYTGYSLLATALALPDDGKILAMDINRENYELGLPVIQKAGVAHKIEFKEGPAMPVLDKLVEDEKNHGSYDFIFVDADKDNYLNYHKRLIELVKVGGLIGYDNTLWNGSVVAPPDAPLRKYVRYYRDFVLELNKALAVDPRIEICMLPVGDGITLCRRVK

>GrCCoAOMT3

MDNEKKRSASFSKGLLQSGELYQYVLETNVYPREPELLKELRDMTATHPRAIMATAPDAGQLIAMLLKLTNAKRTIEVGVFTGYSLLLTAFTIPEDGKIVAIDMNREAYEIGLPVIRKAGVENKIEFIESEALPVLDQLLEDPGNENGFDFAFIDADKINYWKYHERLMKLVKVGGIVVYDNTLWAGSVAMPEECTPEILREGRERTLEFNKLLAADPLVEISLAPLGDGITIVCTELFIQSLNHFKHVCGGSLFNYCISQGIMKLNKESQRPPFSFKCLVLGFRLLLV

>GrCCoAOMT4

MTKSAAKNPTPPDLYLFLQMANIFAINRCSSSVYQRFIVQFNSTQRFSSLSSHVSKSRFIKLNNLRRNCSLSSDAAAPFIVADDEKYGNKQVISITPRLYDYILANAREPPVLRQLREETANMRGSQMQVSPDQAQLLAMLVQILGAARCIELGVYTGYSSLAIALALPESGCLVACERDAK

SLEVAKRYYELAGVSHKVSVKHGLAADVLKSMISNGETCSYDFAFVDAEKRMNQKYFELLLQLVRVGGVIVIDNVLWHGKVADPLVNDAKTVSIRNFNRNLMADDRVSISLVPIGDGMTICRKR

>GrCCoAOMT5

MGPSPTFDQFGTSPTPDRGGKRSNPDFRLHITPLHFFFPLRFPIQRKLKLAQVHKQRKAKRKKLFISRVLAMATNTQDQQSQAGRHQEVGHKSLLQSDALYQYILETSVYPREPEPMKELRKLTAKHPWNLMTTSADEGQFLNMLLKLINAKNTMEIGVYTGYSLLATALAIPDDGKILAMDVNRENYELGLPVIQKAGVAHKIDFKEGPALPVLDQLVEDEKNHGSFDFIFVDADKDNYLNYHKRLIELVKVGGLIGYDNTLWNGSVVAPPDAPLRKYVRYYRDFVMELNKALAVDPRIEICMLPVGDGITLCRRVK

>GrCCoAOMT6

MICFSSIYLKSMEFTTAEPFLHKGLLQSFELTKYILKTNVYPREPSPLKELREVTAKHPGNFMSTTPDSGQLMGMLLKLINAKKTIEIGVYTGYSLLLTALSIPHDAMIIAIDPNKETYEIGLPIIQKAGVEHKINFIESQALPVLDKLLQNLNNEGSFDFAFVDADKENYLNYHERLLKLVKVGGLIVFDNTLWGGTVAQPEEAVSEDRKESRRSIIEFNNSVSIDQRIEIALTPSGDGLTICRRIR

>HcCCoAOMT

MATNTQEQQSQAGRHQEVGHKSLLQSDALYQYILETSVYPREPESMKELRELTAKHPWNLMTTSADEGQFLNMLLKLINAKNTMEIGVYTGYSLLATALALPDDGKILAMDINRENYELGLPVIQKAGVAHKIEFKEGPAMPVLDQLVEDEKNHGSYDFIFVDADKDNYINYHERLIKLVKVGGLIGYDNTLWNGSVVAPPDAPLRKYVRYYRDFVLELNKALAADTRIEICMLPVGDGITLCRRVK

>LuCCoAOMT1

MAAANGVAYRCLAVATATPAISSARCCSSKAWNSIRKVSNLRCSSFKNNPSSTDVVVVAEDEKYGNKQIISLTPRLYDYILSNVREPEIMRQLREETAGMRGSQMQVSPDQAQLLAMLVQLLGAQRCIEVGVYTGYSSLAVALVLPEFGQLIACERDANSLEVANRFYKRAGVDHKVIAKHGMAAEILNSLIQNGESGSYDFAFIDAEKRMNHEYFELLLKLVKVGGVIVIDNVLWHGKVADPLVIDVKTESIRSFNRRIMEDERVSISMVPIGDGMTICRKR

>LuCCoAOMT2

MDLPHKGILQSPDLAKYIYETSVYPREHEQLKKIREATVAKYGNMAETAVPVDEGRFLSLLVKLLNPKRTLEIGVFTGYSLLSTALALSDDSLITAIDISKNHYDIGFPYIKEAGVADKINFIESPATPALEHLLVAIKNEEEALYDFAFVDADKPSYKDYHEHLVKLVKVGGLIAYDNTLWFGFVAKNESEVPERFRGDRKAIMELNKALATDPRVDVAQISVGDGITLCRRIL

>LuCCoAOMT3

MAEEQKSSSENVSRHQEVGHKSLLQSDALYQYILETSVYPREPESMKELREVTAKHPWNIMTTSADEGQFLNMLLKLINAKNTMEIGVYTGYSLLATALAIPDDGKILAMDINRENYEIGLPIIEKAGLAHKIEFREGPALPALDLMVEDKSLHGTYDFIFVDADKDNYINYHKRLIDLVKIGGVIGYDNTLWNGSVVAPPDAPLRKYVRYYRDFVLELNKALAADPRIEICMLPVGDGITLCRRIS

>LuCCoAOMT4

MAEQQQSGENVSRHQEVGHKSLLQSDDLYQYILETSVYPREPESMKELREVTAKHPWNIMTTSADEGQFLNMLLKLINAKNTMEIGVYTGYSLLATALALPDDGKILAMDINRENYEIGLPIIEKAGLAHKIEFKEGPALPALDKMVEDKANHGAYDFIFVDADKDNYINYHKRLIDLVKIGGVIGYDNTLWNGSVVAPPDAPLRKYVRYYRDFVLELNKALAADPRIEICMLPVGDGITLCRRIS

>LuCCoAOMT5

MAEQQQSGENVSRHQEVGHKSLLQSDDLYQYILETSVYPREPESMKELREVTAKHPW

NIMTTSADEGQFLNMLLKLINAKNTMEIGVYTGYSLLATALALPDDGKILAMDINRE

NYEIGLPIIEKAGLAHKIEFKEGPALPALDKMVEDKANHGAYDFIFVDADKDNYINY

HKRLIDLVKVGGVIGYDNTLWNGSVVAPPDAPLRKYVRYYRDFVLELNKALAADPRI

EICMLPVGDGITLCRRIS

>LuCCoAOMT6

MAEEQKQSSSENVSRHQEVGHKSLLQSDALYQYILETSVYPREPESMKELREVTAKHPW

NIMTTSADEGQFLNMLLKLINAKNTMEIGVYTGYSLLATALAIPDDGKILAMDINRENY

EIGLPIIEKAGLAHKIEFREGPALPALDLMVEDKSLHGTYDFIFVDADKDNYINYHKRL

IDLVKIGGVIGYDNTLWNGSVVAPPDAPLRKYVRYYRDFVLELNKALAADPRIEICMLP

VGDGITLCRRIS

>OsCCoAOMT1

MAATGAGEGKETAAVAGGGGGGSLHSKTLLKSEPLYQYVLESTVFPREPDCLRELRLATANHPMAVMAASPDQVQLFGLLIELISAKNAIEVGVFTGYSLLATALALPDDGKIVAIDVSRESYDEVGAPVIDKAGVAHKVDFRVGLAMPVLDELVAEEGNKGRFDFAFVDADKVNFLGYHERLLQLVRVGGLIAYDNTLWGGSVAAPPAAADEAVPSGRDRSLAALAREFNAAIAADRRVKPCQLAIADGVMLCRRVA

>OsCCoAOMT2

MPLLVTLLPVYCTAHSRRLKRTTPASRVSSTAMAAANGDASHGANGGIQIQSKEMKTAIHSNDSPKTLLKSESLHEYMLNTMVYPRENEFMRELRLITSEHTYGFMSSPPEEGQLLSLLLNLTGAKNTIEVGVFTGCSVLATALAIPDDGKVVAIDVSREYFDLGLPVIKKAGVAHKVDFREGAAMPILDNLLANEENEGKFDFAFVDADKGNYGEYHERLLRLVRAGGVLAYDNTLWGGSVALEDDSVLEEFDQDIRRSIVAFNAKIAGDPRVEAVQLPVSDGITLCRRLV

>OsCCoAOMT3

MWGLVDAKLIRVSTAMHRFASASSLPPPAPATAAAAAAQAAALRFGSAATTRVPRALALTASTCPWHRRHLCSSSSSSSSAAAAAATAAAVEEARQGRKQLGATTQLYEYLLANVREHPVLKELREETAAMRGSQMQVSPAQAQLLAMLAQILGAQRCIEVGVYTGYSSLAVALALPESGRLVACERDERCLEVAKKYYQRSGVAHKVDVKHALAADSLKLLIDGGEVNSYDFAFVDADKRMYEEYYELLLKLVRVGGLIVIDNVLWYGRVADPLVNDRKTISIRNFNKKLLEDNRVSISMVPIGDGMTICRKLVDT

>OsCCoAOMT4

MAEAASAAAAATTEQANGSSGGEQKTRHSEVGHKSLLKSDDLYQYILETSVYPREHECMKELREVTANHPWNLMTTSADEGQFLNLLLKLIGAKKTMEIGVYTGYSLLATALAIPDDGTILAMDINRENYELGLPSIEKAGVAHKIDFREGPALPVLDQLVEEEGNHGSFDFVFVDADKDNYLNYHERLMKLVKVGGLVGYDNTLWNGSVVLPADAPMRKYIRYYRDFVLELNKALAADHRVEICQLPVGDGITLCRRVK

>OsCCoAOMT5

MATYRPGSNTLLKSDSILEYVLDTTVYPREHERLRELRLITQNHPKSFMGSSPDQMQFFSVLLKMIGARNAVEVGVFTGYSLLATALALPDDGKVVAIDVSREYYELGRPVIEDAGVAHKVDFRHGDGLAVLDQLLAGGEGKFDFAYADADKEQYRGYHERLVRLLRVGGVVAYDNTLWGGSVAMPRDTPGSSAYDRVVRDYMVGFNAMVAADDRVEACLLPVADGVTLCRRLK

>OsCCoAOMT6

MTTGNGDAPVIKNAHSDIDSTNKTLLKSDALYKYVLDTTVLPREPECMRDLRLITDKHQWGFMQSSADEAQLLGMLLKMAGAKRTIEVGVFTGYSLLATALALPEDGKVVAIDPDRESYEIGRPFLEKAGVAHKVDFREGKGLEKLDELLAEEAAAGREAAFDFAFVDADKPNYVKYHEQLLQLVRVGGHIVYDNTLWAGTVALPPDTPLSDLDRRFSVAIRDLNSRLAADPRIDVCQLAIADGITICRRLV

>TcCCoAOMT1

MDNEKKESTSFSKGLLQSEELYQYILGTSVYPRESEHLKELRDITATHPRAVMATAPDAGQLIALLLKLINAKRTIEVGVFTGYSLLLTALTIPEDGKIVAIDMNREAYEIGLPIIRRAGVDNKIDFIESEALPVLDQLLEDPGNENGFDFAFIDADKINYWNYHERLMKLVKVGGIVVYDNTLWAGTVALSEQSTPEAMREGRQRTLDVNKLLAADSRVQISLAPLGDGITICRRIL

>TcCCoAOMT2

MADTPMKMILRTEALRKYIYETSAYPKEHEQLKELRETTVEKYQKRSFMSTPVDEAQFLSMLVKIMNAKKTMEIGVFTGYSLLTTALALPQDGKILAIDRDKEAYEFGLPYIKKAGIEHKINFVASDAISALNDLVNSGEEGTFDFIFVDALKSEYLKYHELTMKFVKIGGVIAYDNTLWSGSVAQAEDQVEMPGVSISELLNLFQELRIVLLPDESE

>TcCCoAOMT3

MLYLFPFYNLLVGFNLPFNGEDIFQEFRSMADTPMKMILRTEALRKYIYETSAYPKEHEQLKELRETTVEKYQKRSFMSTPVDEAQFLSMLVKIMNAKKTMEIGVFTGYSLLTTALALPQDGKILAIDRDKEAYEFGLPYIKKAGIEHKINFVASDAISALNDLVNSGEEGTFDFIFVDALKSEYLKYHELTMKFVKIGGVIAYDNTLWSGSVAQAEDQVEMPGIRSFRKFVIEFNSFIAADPRVESSLLSIGDGVTLCRRLY

>TcCCoAOMT4

MANMNTSSKGLLKSEALKKYILNTSAYPREHEQLKGIRDATAQKYPNLCAMGVPVDEGQFLSMLLKLMNAKRTMEIGVFTGYSLLATALALPDDGKITAIDTSWEFFEVGLPFIKKAGMEHKINFIESDAMKVLNEMSSNDKQKPEFDFVFVDADKTSYMKYHEHIKKLVKIGGVVAYDNTLWFGFLAQEEAEVPESARASRKAMLNFNVNLASDPCMEVSQVSIGDGVTLCRRIS

>TcCCoAOMT5

MAVSSLKYKSLIVHRPILEYILEKNAYPKEHEQLKELREATAEKYGKKSIMNVPADEAQFLSMLLKVMNAKKTLEIGVFTGYSLLATALALPDDGQITAIDLDKEAYETGLPFIKKAGVEHKINFVHSDAFLVLNDLINGENEETLDFIFVDAEKKDYMKLHEQVLKLVKVGGIIAYDNTLWFGSVAYEEEKDEMPEFVWGTREYVVQFNSFLAADPRIELSLLSIGDGVTLCRRLH

>TcCCoAOMT6

MATNTQEQQSQAGRHQEVGHKSLLQSDALYQYILETSVYPREPEPMKELRELTAKHPWNLMTTSADEGQFLNMLLKLINAKNTMEIGVYTGYSLLATALALPDDGKILAMDINRENYELGLPVIQKAGVAHKIDFKEGPAMPALDQLVEDEKNHGSFDFVFVDADKDNYINYHKRLIELVKVGGLIGYDNTLWNGSVVAPPDAPLRKYVRYYRDFVLELNKALAADPRIEICMLPVGDGITLCRRVK

>TcCCoAOMT7

MAPTQEGQQNQAGRHQEVGHKSLLQSDNLYQYILETSVYPREPEPMKELRELTAKHPWNIMTTSADEGQFLNMLLKLINAKNTMEIGVYTGYSLLATALALPDDGKILAMDINRENYELGLPVIQKAGVAHKIDFKEGPSLPVLDQMIEAGKYHGTFDFIFVDADKDNYLNYHKRLIELVKVGGVIGYDNTLWNGSVVAPPDAPLRKYVLYYRDFVLELNRALAADPRIEICQLPVGDGITLCRRIS
