# Supplementary material for: Evolution and expression analysis of the caffeoyl-CoA 3-O-methyltransferase (CCoAOMT) gene family in jute (Corchorus L.)
Source: BMC Genomics. 2023 Apr 17;24:204. doi: 10.1186/s12864-023-09281-w (PMC10111781; doi:10.1186/s12864-023-09281-w)
Supplement: Supplementary file 8 — Additional file 8. A schematic diagram for the relationship of the 5 groups of the phylogenetic tree constructed by NJ method. [file 12864_2023_9281_MOESM8_ESM.pdf]

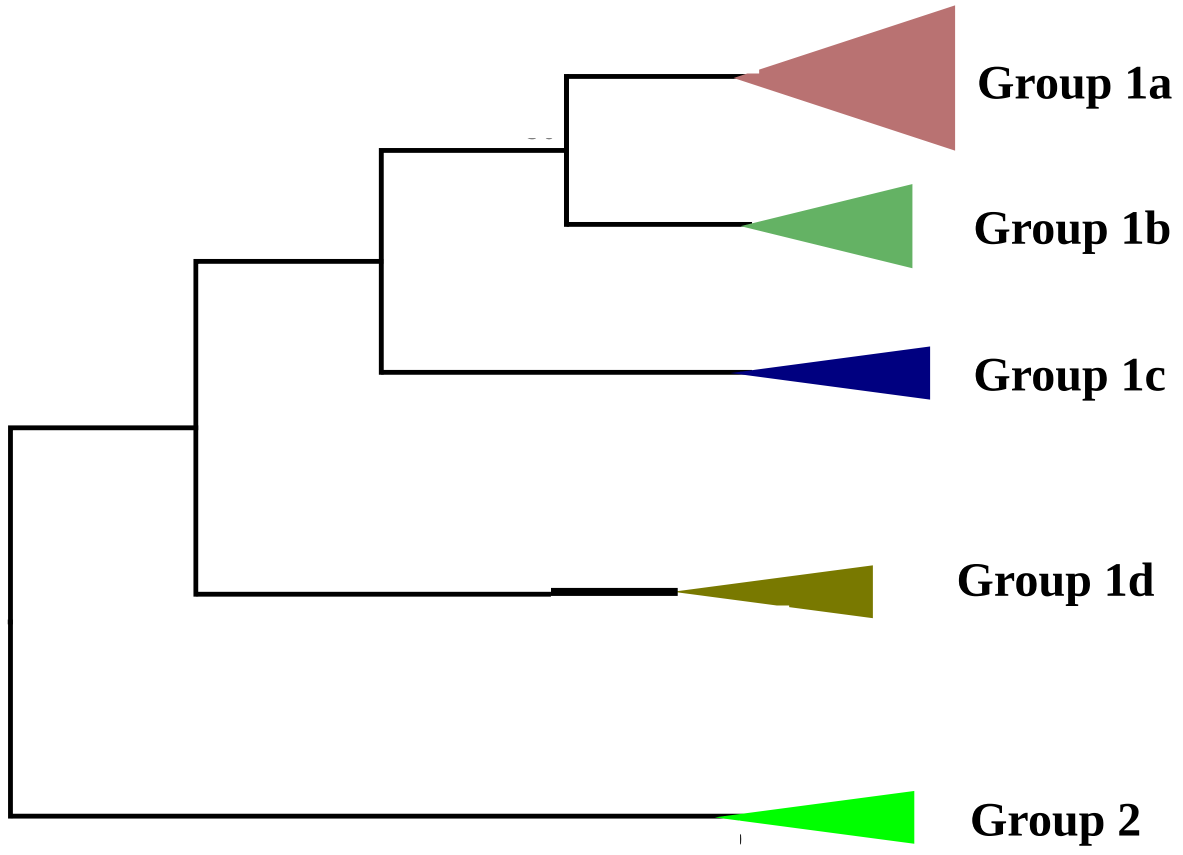

Additional file 8: A schematic diagram for the relationship of the 5 groups of the phylogenetic tree constructed by NJ method.
