# Supplementary material for: Evolution and expression analysis of the caffeoyl-CoA 3-O-methyltransferase (CCoAOMT) gene family in jute (Corchorus L.)
Source: BMC Genomics. 2023 Apr 17;24:204. doi: 10.1186/s12864-023-09281-w (PMC10111781; doi:10.1186/s12864-023-09281-w)
Supplement: Supplementary file 9 — Additional file 9. Divergence time among the five groups of CCoAOMT gene family in jute. [file 12864_2023_9281_MOESM9_ESM.docx]

**Additional file 9: Divergence time among the five groups of *CCoAOMT* gene family in jute.**

| Group-Group | Median Ks | Gene pairs used | Divergence time (Mya) |
| --- | --- | --- | --- |
| 1a-1b | 2.53528 | 12 | 207.8 |
| 1a-1c | 4.09807 | 12 | 335.9 |
| 1a-1d | 2.10191 | 28 | 172.3 |
| 1a-2 | 2.555205 | 8 | 209.4 |
| 1b-1c | 2.403902 | 9 | 197 |
| 1b-1d | 2.438115 | 21 | 199.8 |
| 1b-2 | 3.40588 | 6 | 279.2 |
| 1c-2 | 1.88155 | 21 | 154.2 |
| 1d-2 | 2.24837 | 14 | 184.3 |
