# Supplementary material for: Evolution and expression analysis of the caffeoyl-CoA 3-O-methyltransferase (CCoAOMT) gene family in jute (Corchorus L.)
Source: BMC Genomics. 2023 Apr 17;24:204. doi: 10.1186/s12864-023-09281-w (PMC10111781; doi:10.1186/s12864-023-09281-w)
Supplement: Supplementary file 13 — Additional file 13. Overall collinearity relationships of CCoAOMT genes on the jute genome. [file 12864_2023_9281_MOESM13_ESM.docx]

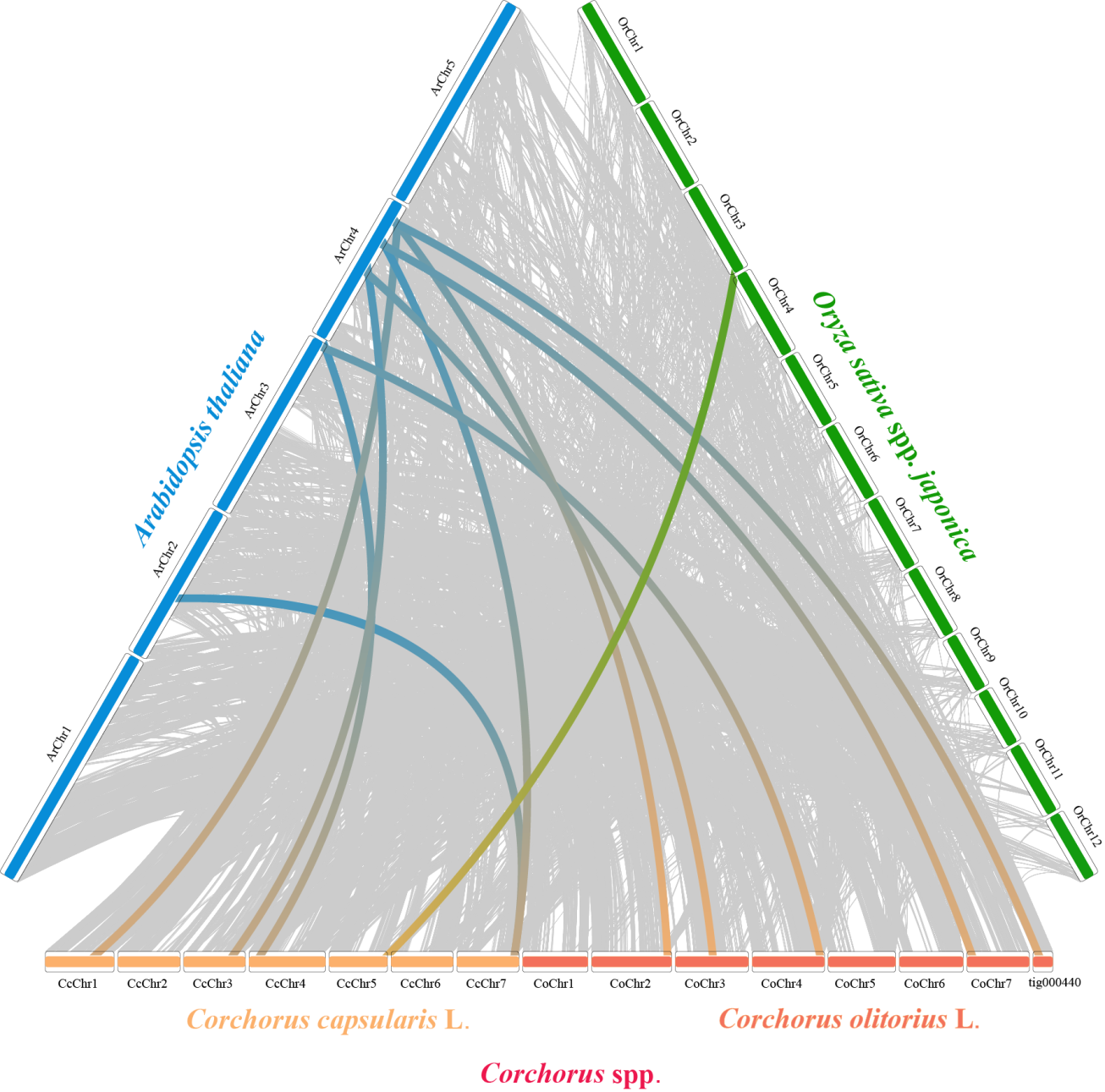


Additional file13: Overall collinearity relationships of *CCoAOMT* genes on the jute genome. Collinearity of jute *CCoAOMT* genes were compared to their counter parts in Arabidopsis and rice.
