# Supplementary material for: Evolution and expression analysis of the caffeoyl-CoA 3-O-methyltransferase (CCoAOMT) gene family in jute (Corchorus L.)
Source: BMC Genomics. 2023 Apr 17;24:204. doi: 10.1186/s12864-023-09281-w (PMC10111781; doi:10.1186/s12864-023-09281-w)
Supplement: Supplementary file 14 — Additional file 14. Expression profile of CCoAOMT2 in six cultivars of jute at each developmental stage. [file 12864_2023_9281_MOESM14_ESM.docx]

| A | B |
| --- | --- |
|  |  |
| C | D |
|  |  |
| E |  |
|  |  |

**Additional file 14: Expression profile of *CCoAOMT2* in six cultivars of jute at each developmental stage.**

**A: Comparison of the six cultivars of jute at 15 DAS. B: Comparison of the six cultivars of jute at 30 DAS. C: Comparison of the six cultivars of jute at 45 DAS. D: Comparison of the six cultivars of jute at 60 DAS. E: Comparison of six cultivars of jute at 90 DAS.**

**Note: SDs and mean values were acquired from three biological replicates. The character S refers to stage. Turkey test was used for statistical analysis, and different letters expressed significant differences between cultivars at each stage (p<0.05).**
