# Supplementary material for: Evolution and expression analysis of the caffeoyl-CoA 3-O-methyltransferase (CCoAOMT) gene family in jute (Corchorus L.)
Source: BMC Genomics. 2023 Apr 17;24:204. doi: 10.1186/s12864-023-09281-w (PMC10111781; doi:10.1186/s12864-023-09281-w)
Supplement: Supplementary file 15 — Additional file 15. Change in expression level of CCoAOMT genes that were highly expressed in different stages of stem development in jute. [file 12864_2023_9281_MOESM15_ESM.pdf]

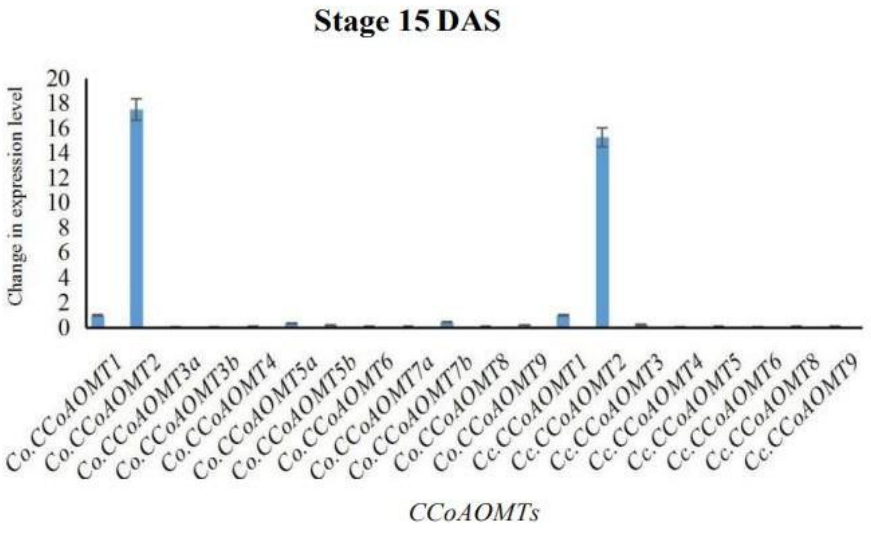
Stage 15 DAS
Change in expression level
20
18
16
14
12
10
8
6
4
2
0
Co.CCoAOMT1
Co.CCoAOMT2
Co.CCoAOMT3a
Co.CCoAOMT3b
Co.CCoAOMT4
Co.CCoAOMT5a
Co.CCoAOMT5b
Co.CCoAOMT6
Co.CCoAOMT7a
Co.CCoAOMT7b
Co.CCoAOMT8
Co.CCoAOMT9
Cc.CCoAOMT1
Cc.CCoAOMT2
Cc.CCoAOMT3
Cc.CCoAOMT4
Cc.CCoAOMT5
Cc.CCoAOMT6
Cc.CCoAOMT8
Cc.CCoAOMT9
CCoAOMTs

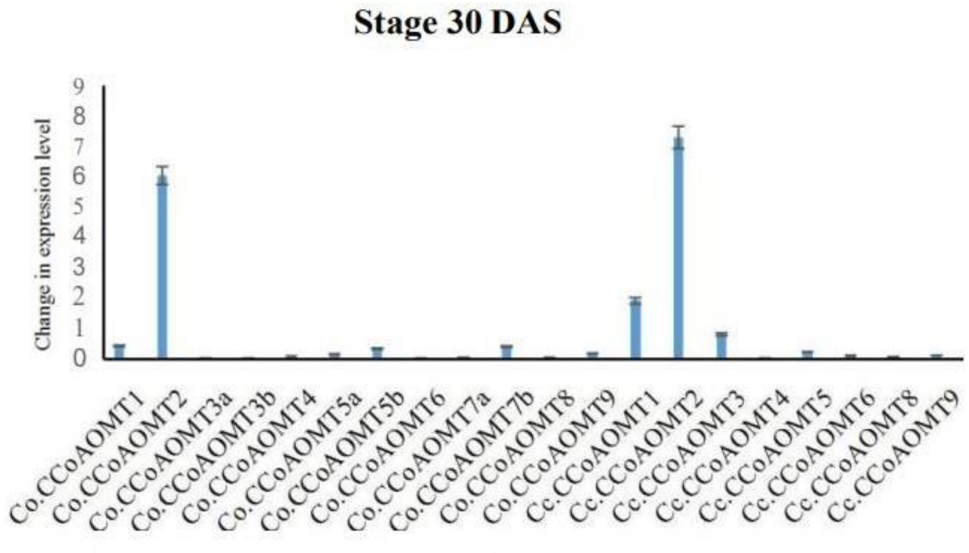
Stage 30 DAS
Change in expression level
9
8
7
6
5
4
3
2
1
0
Co.CCoAOMT1
Co.CCoAOMT2
Co.CCoAOMT3a
Co.CCoAOMT3b
Co.CCoAOMT4
Co.CCoAOMT5a
Co.CCoAOMT5b
Co.CCoAOMT6
Co.CCoAOMT7a
Co.CCoAOMT7b
Co.CCoAOMT8
Co.CCoAOMT9
Cc.CCoAOMT1
Cc.CCoAOMT2
Cc.CCoAOMT3
Cc.CCoAOMT4
Cc.CCoAOMT5
Cc.CCoAOMT6
Cc.CCoAOMT8
Cc.CCoAOMT9
CCoAOMTs

Stage 45 DAS

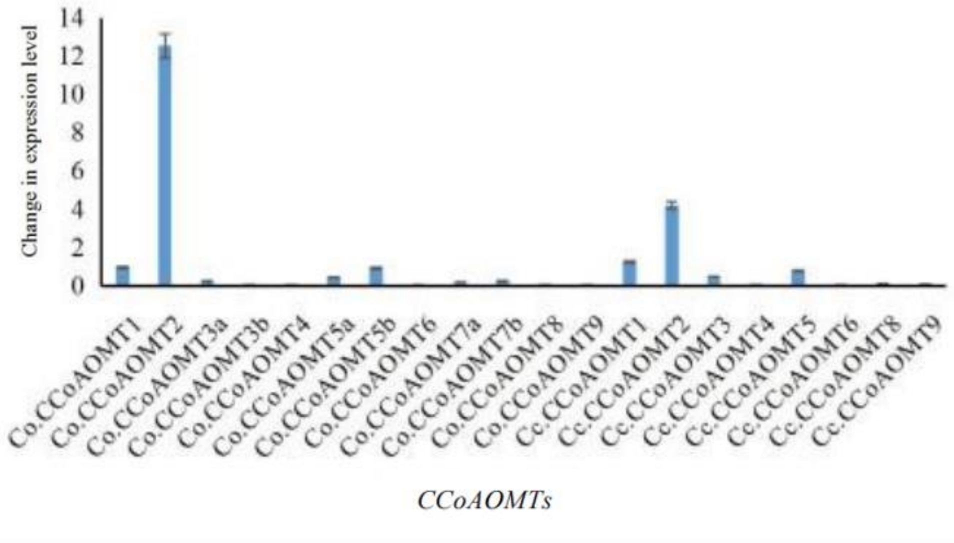

Stage 60 DAS

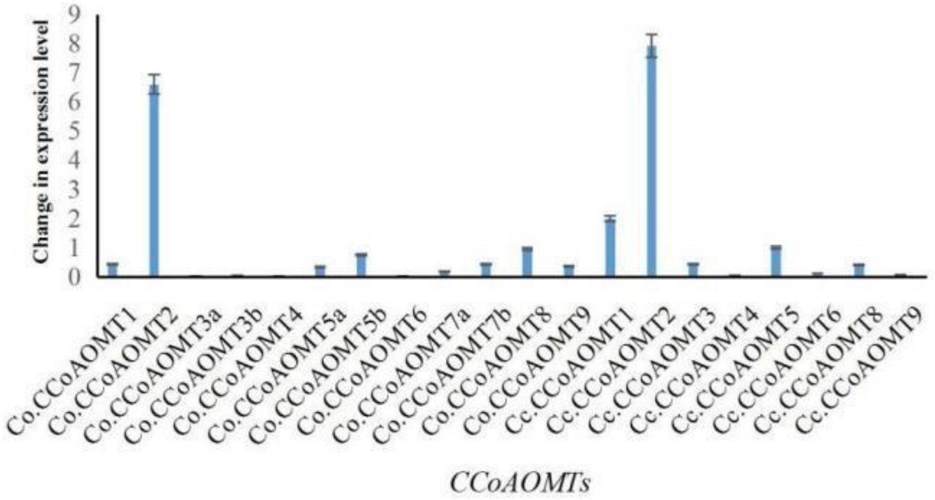

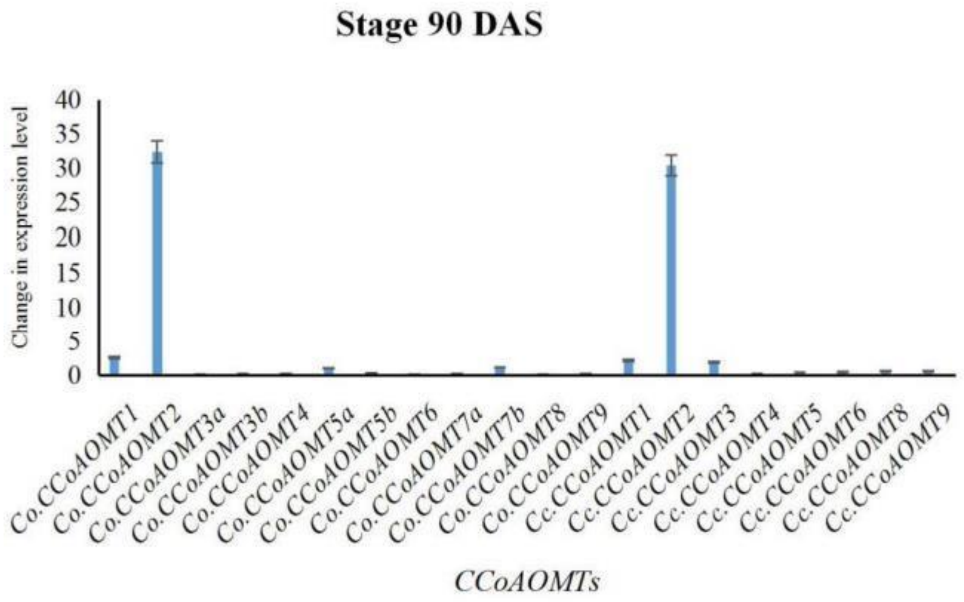

Additional file15: Change in expression level of *CCoAOMT* genes that were highly expressed in different stages of stem development in jute. The expression of all *CCoAOMTs* were normalized to the lowest expressing *CCoAOMT* in each stage separately.
