# Supplementary material for: Evolution and expression analysis of the caffeoyl-CoA 3-O-methyltransferase (CCoAOMT) gene family in jute (Corchorus L.)
Source: BMC Genomics. 2023 Apr 17;24:204. doi: 10.1186/s12864-023-09281-w (PMC10111781; doi:10.1186/s12864-023-09281-w)
Supplement: Supplementary file 16 — Additional file 16. The expression levels of CCoAOMT genes under different parts (top, middle, bottom) of 60 DAS of the stem of C. capsularis and C. olitorius. [file 12864_2023_9281_MOESM16_ESM.pdf]

A

Co.CCoA OMTs	top	middle	bottom
Co.CCoA OMT1	1	3.702308408	2.341767363
Co.CCoA OMT2	25.7004819	29.08893979	15.68009176
Co.CCoA OMT3a	0.033060953	0.139974532	0.076809746
Co.CCoA OMT3b	0.040151436	0.104896729	0.062280226
Co.CCoA OMT5a	6.116417964	1.819486304	2.377094054
Co.CCoA OMT5b	2.149030374	1.695872871	4.348616322
Co.CCoA OMT6	0.177853022	0.387050543	0.218872086
Co.CCoA OMT7a	0.556640923	2.583047834	6.151258586
Co.CCoA OMT7b	2.169645605	2.327664382	4.509315697
Co.CCoA OMT8	0.232512276	0.607550392	0.333750882
Co.CCoA OMT9	0.777471796	0.831101459	1.07862203

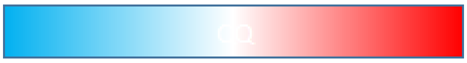

B

Cc.CCoA OMTs	top	middle	bottom
Cc.CCoA OMT1	1	2.510951068	3.714771925
Cc.CCoA OMT2	6.43434106	11.90111973	20.50228457
Cc.CCoA OMT3	0.575273248	0.435418045	0.281229711
Cc.CCoA OMT4	0.224940435	0.014022411	0.006106107
Cc.CCoA OMT5	0.39248965	2.194941512	0.341062067
Cc.CCoA OMT6	0.09711839	0.023437704	0.021769662
Cc.CCoA OMT8	0.297095644	0.101936747	0.145035437
Cc.CCoA OMT9	0.281637814	0.168007423	0.168553463

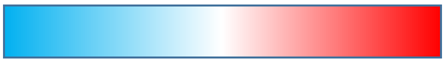

Additional file16: The expression levels of *CCoAOMT* genes under different parts (top, middle, bottom) of 60 DAS of the stem of *C. capsularis and C. olitorius.*

Note: A. The transcript levels of *CCoAOMT* members in the stem of *C. olitorius*.

B. The expression patterns of *CCoAOMT* genes in *C. capsularis.*
