# Supplementary material for: Evolution and expression analysis of the caffeoyl-CoA 3-O-methyltransferase (CCoAOMT) gene family in jute (Corchorus L.)
Source: BMC Genomics. 2023 Apr 17;24:204. doi: 10.1186/s12864-023-09281-w (PMC10111781; doi:10.1186/s12864-023-09281-w)
Supplement: Supplementary file 17 — Additional file 17. The sum of normalized expression of all CCoAOMT genes from each developmental stage. [file 12864_2023_9281_MOESM17_ESM.pdf]

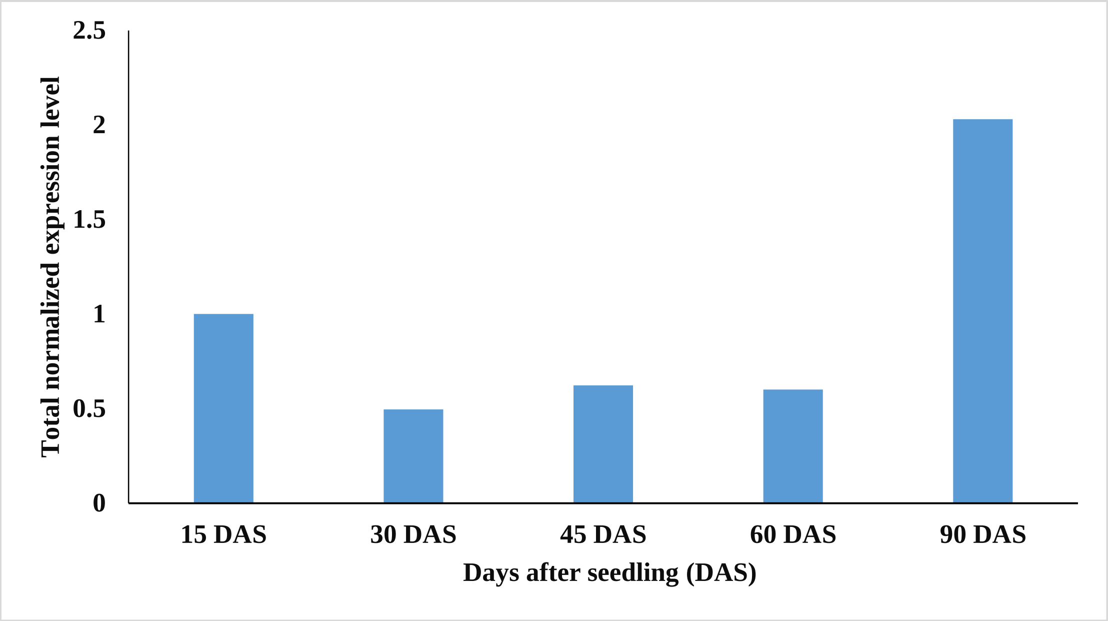

Additional file 17: The sum of normalized expression of all *CCoAOMT* genes from each developmental stage. Expression level of all *CCoAOMT* genes from each developmental stage were added showing a maximum expression at 90 DAS. Actin7 was used as an internal control to normalize the expression data of each *CCoAOMT*. 15 DAS was set to be 1 and expression level at other stages was calculated accordingly.
