# Supplementary material for: Evolution and expression analysis of the caffeoyl-CoA 3-O-methyltransferase (CCoAOMT) gene family in jute (Corchorus L.)
Source: BMC Genomics. 2023 Apr 17;24:204. doi: 10.1186/s12864-023-09281-w (PMC10111781; doi:10.1186/s12864-023-09281-w)
Supplement: Supplementary file 19 — Additional file 19. PCR primer sequences of CCoAOMT genes in jute. [file 12864_2023_9281_MOESM19_ESM.pdf]

Additional file19: PCR primer sequences of *CCoAOMT* genes in jute.

Genes	Forward and Reverse primers
Co.CCoAOMT1-F	CATGAAAAAGTGTGTTTAATGATGAGT
Co.CCoAOMT1-R	TCCGACTCTTCCCAACGAGT
Co.CCoAOMT2-F	TAAATGTATCCTCAAAAGTGACGCTCT
Co.CCoAOMT2-R	CCAATTGAAATCTGACAAACCTCCATG
Co.CCoAOMT3a-F	TGGCTACTGCTCCTGATGCG
Co.CCoAOMT3a-R	CAGTGAACACGCCTGCAG
Co.CCoAOMT3b-F	AGCAAACTCAAGCTAGTAGGCAC
Co.CCoAOMT3b-R	GTAATTCCATCGCCAACAGGAAGTT
Co.CCoAOMT4-F	TGCTCTCCAAGAGGTATATAAATTTCTC
Co.CCoAOMT4-R	TAATAAAGGCGCCTGCAGAGAG
Co.CCoAOMT5a-F	AGTATGGAAAATACCAGTGATCAACAA
Co.CCoAOMT5a-R	AGTAGCCAGTATAAACACCAATTTCAA
Co.CCoAOMT5b-F	TGGCAGAGGCAGTCTCCAG
Co.CCoAOMT5b-R	TAATAGAGTCGCCTGCAGAGG
Co.CCoAOMT6-F	TCAACTTTATTCCCAGTCGCAAATATC
Co.CCoAOMT6-R	GCAGCCAAATGCAGGTATCAATTT
Co.CCoAOMT7a-F	TGGCTGATTGGTTTAAGAAGACG
Co.CCoAOMT7a-R	TAAATGTAGAAGTTTACCTTGCCATCCT
Co.CCoAOMT7b-F	TGGCAGAGGCAGTCTCCA
Co.CCoAOMT7b-R	AAAGCTTGTTACATAATGTTAAAGGCC
Co.CCoAOMT8-F	TGAATGTGCCTGCAGATGAAG
Co.CCoAOMT8-R	TAGTAAAGACGCCTGCAGAGAG
Co.CCoAOMT9-F	CATTTGATTCGACGGCAAAGG
Co.CCoAOMT9-R	TGGCAACCAATACCCAAGAGC
Cc.CCoAOMT1-F	TGGCTCCAACTCAGGCAG
Cc.CCoAOMT1-R	CACTTAATCCGACGGCAGAG
Cc.CCoAOMT2-F	GGATCCAAAAAGGTGGGGAGAATTTA
Cc.CCoAOMT2-R	CAGCTTATGATTATAGGCGGATGATCC
Cc.CCoAOMT3-F	CACAAATTAAAAAAACAGAGAAGGAA
Cc.CCoAOMT3-R	CATTTGATCCGACGGCAAAGG
Cc.CCoAOMT4-F	TGAGTTCCGACTCATCCCAAC
Cc.CCoAOMT4-R	CATGAAAAAATGTGTTTAATGATGAGT
Cc.CCoAOMT5-F	CACAAGCATGTTTCGGAGGC
Cc.CCoAOMT5-R	TGCATTCGGCTCCTCCCTTAT
Cc.CCoAOMT6-F	CTTCAAATATTCATGGTATTTCATGTAA
Cc.CCoAOMT6-R	CATAAAATCAATTTTATAGACTCTGAA
Cc.CCoAOMT8-F	TCAGTGAAGACGCCTGCAGATTGTAAT
Cc.CCoAOMT8-R	GGACCTCCAGTGTCCTCACAGATG
Cc.CCoAOMT9-F	GAGGCCATCGCCAATAGAAAGAAGA
Cc.CCoAOMT9-R	TCCTTAGAAATGATGCCCTTGTTCAGG
