# Supplementary material for: Evolution and expression analysis of the caffeoyl-CoA 3-O-methyltransferase (CCoAOMT) gene family in jute (Corchorus L.)
Source: BMC Genomics. 2023 Apr 17;24:204. doi: 10.1186/s12864-023-09281-w (PMC10111781; doi:10.1186/s12864-023-09281-w)
Supplement: Supplementary file 20 — Additional file 20. Jute CCoAOMT gene-specific primers and reference genes used for qRT-PCR analysis. [file 12864_2023_9281_MOESM20_ESM.pdf]

Additional file 20: Jute *CCoAOMT* gene-specific primers and reference genes used for qRT-PCR analysis.

Genes	Forward primer and Reverse primer (5'-3')
Co.CCoAOMT1-F	*CAGCTGATGAAGGGCAGTTT*
Co.CCoAOMT1-R	*GCAGTGGCAAGGAGTGAATAG*
Co.CCoAOMT2-F	*TTCGTGGATGCTGACAAAGA*
Co.CCoAOMT2-R	*ACAACAGAGCCATTCCATAGAG*
Co.CCoAOMT3a-F	*CACCAGATGCAGGGCAATTA*
Co.CCoAOMT3a-R	*AGCAGTGAGGAGAAGAGAGTAG*
Co.CCoAOMT3b-F	*GTGAAGGTAGGTGGGATTGTT*
Co.CCoAOMT3b-R	*TCTGCCTACCCTCTCTCATTAT*
Co.CCoAOMT4-F	*CAGTAGACGAAGGGCAGTTT*
Co.CCoAOMT4-R	*GCAGTTGCAAGCAAAGAGTAG*
Co.CCoAOMT5a-F	*GTTGGATACGAGGAGGAGAAAG*
Co.CCoAOMT5a-R	*TGCGAGGATCAGTTACTAAGAAG*
Co.CCoAOMT5b-F	*GTCAGTTGGATACGAGGAAGAG*
Co.CCoAOMT5b-R	*GCGAGGATCAGTTGCTAAGA*
Co.CCoAOMT6-F	*TCTTACTACTGCCCTTGCTTTG*
Co.CCoAOMT6-R	*GTGCTCAACTCCAGCTTTCT*
Co.CCoAOMT7a-F	*CCTGCAGATGAAGCACAATTTC*
Co.CCoAOMT7a-R	*GGCAGTAGCAAGAAGGGAATAG*
Co.CCoAOMT7b-F	*ACGAGTGCTTATCCCAAAGAG*
Co.CCoAOMT7b-R	*CCCTTCATCAGCAGGAACAT*
Co.CCoAOMT8-F	*AGATTCTGGGAGCTGAAAGATG*
Co.CCoAOMT8-R	*TCCCTTTCACAGGCAACTAAA*
Co.CCoAOMT9-F	*GCCGTTGCTTAGGAGAGTTAG*
Co.CCoAOMT9-R	*CCACACTGCCAGATAGAGAAAG*
Cc.CCoAOMT1-F	*AGCTGATGAAGGGCAGTTT*
Cc.CCoAOMT1-R	*GGCAGTAGCAAGGAGAGAATAG*
Cc.CCoAOMT2-F	*GGTGTTTACACTGGCTACTCTC*
Cc.CCoAOMT2-R	*GATAACAGGCAGACCCAACTC*
Cc.CCoAOMT3-F	*GTGAAGAGGAGGTGGAGAAATC*
Cc.CCoAOMT3-R	*AGCTAACTGTGGAGTGGAAATAC*
Cc.CCoAOMT4-F	*GTGCATATCCTAGAGAGCATGAG*
Cc.CCoAOMT4-R	*CCCTTCGTCTACAGGTACATTC*
Cc.CCoAOMT5-F	*GAAGGGCAGTTCCTGTCTATG*
Cc.CCoAOMT5-R	*AACGCAAGAGCGGTAGTAAG*
Cc.CCoAOMT6-F	*CTTACTACTGCCCTCGCTTTAC*
Cc.CCoAOMT6-R	*GTGCTCAACTCCAGCTTTCT*
Cc.CCoAOMT8-F	*ATTCGGCTCCTCCCTTATCT*
Cc.CCoAOMT8-R	*GCATCGTTGCGTCCTACTT*
Cc.CCoAOMT9-F	*GCCGTTGCTTAGGAGAGTTAG*
Cc.CCoAOMT9-R	*CCACACTGCCAGATAGAGAAAG*

Reference genes used for the qPCR analysis

**Reference genes**	**Forward and Reverse (5'-3')**
Ubiquitin (UBI)-F	CCACTCTCCACCTTGTCCTC
Ubiquitin (UBI)-R	CAGCCTCTGAACCTTTCCAG
Actin 7 (ACT7)-F	ACAATTGGAGCAGAGCGTTT
Actin 7 (ACT7)-R	TAGACCCACCGCTAAGCACT
Ubiquitin-conjugating (UBC)-F	CTGCCATCTCCTTTTTCAGC
Ubiquitin-conjugating (UBC)-R	CGAGTGTCCGTTTTCATTCA
